# Antifibrotic Efficacy of a Nintedanib–Peptide Conjugate and Diagnostic Potential of a Fluorescent Companion Probe Targeting α_V_β_6_ Integrin in Idiopathic Pulmonary Fibrosis

**DOI:** 10.1021/acsptsci.5c00457

**Published:** 2025-09-17

**Authors:** Kelly Bugatti, Erica Ferrini, Margherita Restori, Costanza Bonfini, Melissa Marchese, Francesca Bianchini, Sara Tomassetti, Andrea Maurizio, Monica Baiula, Lucia Battistini, Enrico Marcantonio, Claudio Curti, Monica Civera, Laura Belvisi, Andrea Sartori, Franco F. Stellari, Franca Zanardi

**Affiliations:** † Department of Food and Drug, 9370 University of Parma, 43124 Parma, Italy; ‡ Molecular Imaging Facility, Experimental Pharmacology & Translational Science Department, 18869 Chiesi Farmaceutici S.p.A., 43122 Parma, Italy; § ANTHEM (AdvaNced Technologies for Human-centrEd Medicine), Spoke 3, 20126 Milan, Italy; ∥ Department of Veterinary Science, University of Parma, 43126 Parma, Italy; ⊥ Department of Experimental and Clinical Biomedical Sciences “Mario Serio”, 9300 University of Florence, 50134 Florence, Italy; # Department of Clinical and Experimental Medicine, University of Florence, 50134 Florence, Italy; ∇ Department of Pharmacy and Biotechnology, 9296 University of Bologna, 40126 Bologna, Italy; ○ Department of Chemistry, 9304 University of Milan, 20133 Milan, Italy

**Keywords:** drug delivery, peptide–drug conjugates, pulmonary fibrosis, optical imaging, RGD peptides, targeted therapy

## Abstract

Idiopathic pulmonary fibrosis (IPF) is a fatal fibrotic pathology currently treated with two antifibrotic drugs, nintedanib and pirfenidone; however, more effective and safer cell-specific therapeutic agents are needed to overcome their limited efficacy and tolerability. αvβ6 integrin is a clinically validated fibrosis biomarker, and several αvβ6-targeted small molecules and positron emission tomography (PET) tracers have recently proven their therapeutic and diagnostic potential in IPF. Surprisingly, αvβ6-targeted and fibrosis-related drug conjugates are still lacking. Two molecular conjugates, namely the previously reported peptide–drug conjugate (PDC) **1** and the novel fluorescent probe **2**, were developed here, where a αvβ6-targeted cyclopeptide is covalently linked to either nintedanib or the near-infrared (NIR) ZW800-1 fluorescent tag via robust linkers. Chemical synthesis of the two compounds, molecular docking studies of **1** in complex with αvβ6, mouse and human plasma stability measurement, binding affinity evaluation toward the isolated αvβ6 receptor, and *in vitro* human IPF-derived fibroblast cell internalization and antifibrotic studies were performed. Then, *in vivo* and *ex vivo* assessments of the antifibrotic efficacy of **1** and the diagnostic potential of **2** were carried out in a bleomycin (BLM)-induced lung fibrosis mouse model. Conjugate **1** demonstrated superior antifibrotic efficacy as compared to the separated peptide and drug components, and probe **2** specifically accumulated in the fibrotic lesions of mice lungs. The molecular conjugates **1** and **2** represent a promising theranostic couple for lung fibrosis and αvβ6-related pathologies and a useful proof-of-principle tool testifying how the simultaneous cell-targeted inhibition of multiple fibrosis-related receptors could be more impactful than the inhibition of one sole receptor.

## Graphical abstract

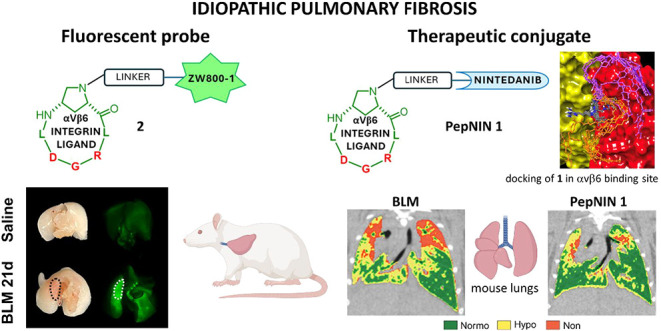

Idiopathic pulmonary fibrosis (IPF)—the most common subtype of pulmonary fibrosis—is a chronic, progressive, and fatal fibrotic pathology characterized by the aberrant accumulation of fibrotic tissues in the lungs, which leads to progressive dyspnea and loss of lung function, and ultimately causing irreversible destruction of lung architecture and respiratory failure.^1–8^ Though, by definition, IPF is a disease of unknown cause, recent studies indicate that repeated alveolar epithelial cell microinjuries like particulates or chemicals are possible triggers of the disease, by sustaining an aberrant epithelial–mesenchymal interaction in susceptible individuals. The subsequent evolution of the fibrotic alterations is crucially driven by the interaction of injured alveolar epithelial cells with a multitude of profibrotic mediators and signaling pathways, including transforming growth factor-β (TGF-β), fibroblast growth factor-2 (FGF-2), and platelet-derived growth factor (PDGF). In addition, it has been shown that receptor tyrosine kinases such as the PDGF receptor (PDGFR), FGF receptor (FGFR), and vascular growth factor receptor (VEGFR) are crucial in stimulating fibroblast activation and extracellular matrix (ECM) synthesis.^1^ In the past decade, two antifibrotic medications were approved worldwide for IPF treatment, namely, nintedanib (Figure 1A) and pirfenidone. Though they have different mechanisms of action and safety profiles, they share similar ability in reducing the pulmonary functional decline and delaying the rate of disease progression; however, neither compound is able to halt fibrosis nor significantly improve the overall survival.^4,5^ In particular, nintedanib is a multitargeted tyrosine kinase inhibitor (TKI) that competitively binds to the intracellular kinase domains of VEGFR-1/2/3, FGFR-1/2/3, and PDGFRα/β; it inhibits fibroblast proliferation, migration, and differentiation, as well as secretion and deposition of ECM in the lungs, hence playing a role in mitigating aggravation of IPF.^9,10^ Nintedanib is a cell-unselective small molecule with poor bioavailability, and approximately 20–25% IPF patients treated with this drug (and with pirfenidone as well) are not able to tolerate the treatment due to severe adverse reactions.^11,12^ The nonresolutive character of the treatment with nintedanib or pirfenidone, the complexity of their clinical management in a fragile patient population, and their poor long-term tolerability all highlight the urgent need to identify novel effective and safer therapeutic agents and strategies for IPF. As mentioned above, dysfunctional epithelial and endothelial cells secrete fibrogenic mediators such as TGF-β, which induce epithelial-to-mesenchymal transition (EMT), as well as fibroblast recruitment, proliferation, and differentiation to myofibroblasts, the main collagen-producing cells. TGF-β is secreted in an inactive form, and its αvβ6 integrin-mediated activation ensures innate, localized anti-inflammatory surveillance, immunosuppression regulation, and tumor suppression.^13^

**Figure 1:**
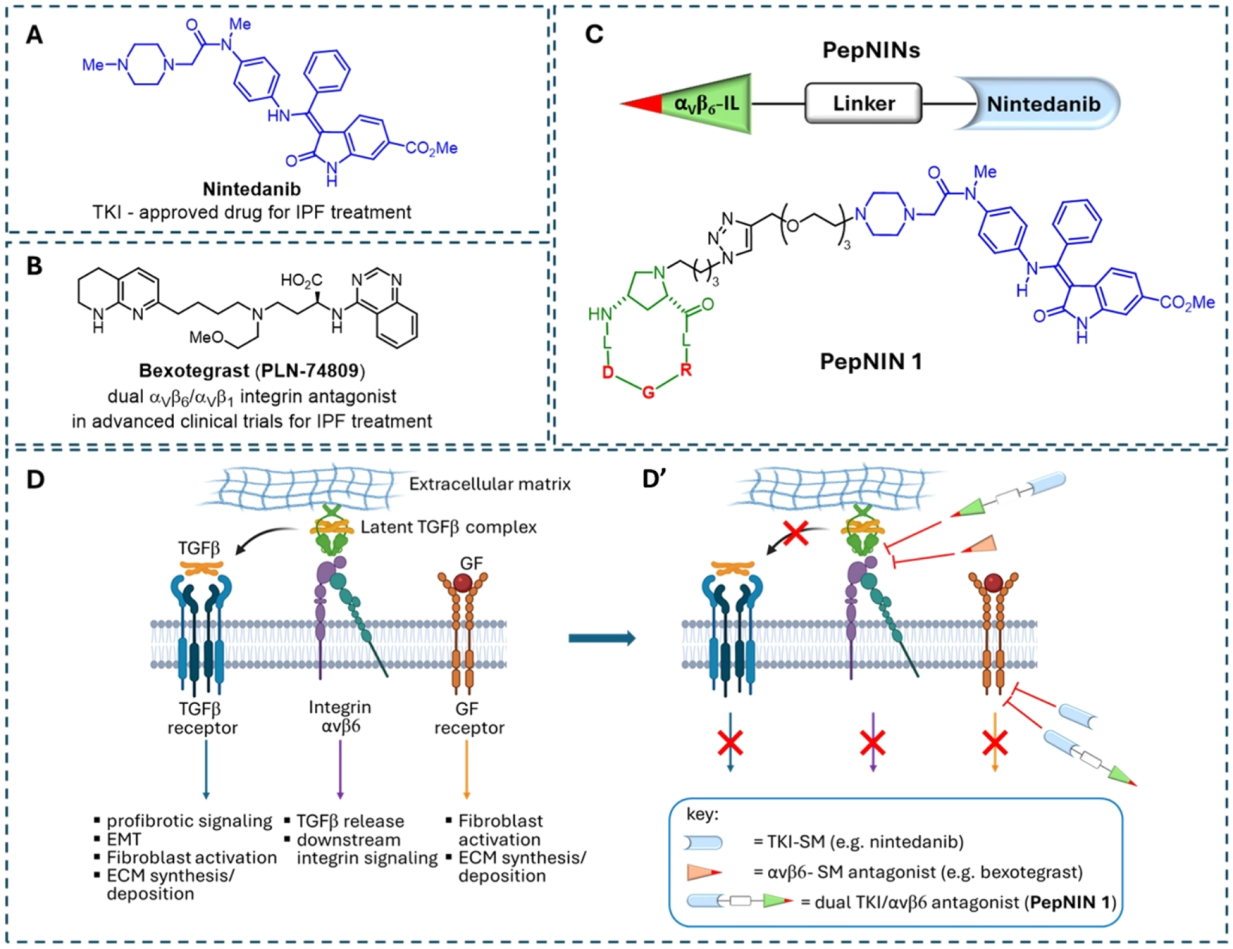
(A) Structure of the small-molecule nintedanib, a multikinase inhibitor approved for treatment against IPF; (B) structure of the small-molecule bexotegrast, a dual αvβ6/αvβ1 integrin antagonist under advanced clinical trials against IPF; (C) representation of the general design of peptide–nintedanib conjugates (PepNINs), and structure of PepNIN **1**, focus of this work; (D) schematic representation of the main players in pulmonary fibrosis in lung epithelial and/or lung fibroblast cells: the TGF-β receptor, αvβ6 integrin receptor, and growth factor receptors (e.g., PGFR, VEGFR, and FGFR), and biological response upon their binding with the respective endogenous ligands TGF-β, latency-associated peptide (LAP), and GF; (D′) depiction of intracellular inhibition of GFR by cell-unselective small-molecule nintedanib with subsequent GF signaling inhibition (*one target-type → one action*); targeted extracellular inhibition of αvβ6 integrin/LAP interaction by the small-molecule bexotegrast with subsequent inhibition of integrin αvβ6 and TGF-β signaling (*one target → two actions*), and targeted extracellular and intracellular dual inhibition of both GFR and αvβ6 integrin by PepNIN **1** of this work with possible subsequent multiple TGF-β/αvβ6 integrin/GFR signaling inhibition (*two targets → multiple actions*). (D, D′) were partially created in BioRender. Sartori, A. (2025) https://BioRender.com/bgtwwwo.

Integrin αvβ6 is a heterodimer belonging to the wide family of Arg-Gly-Asp (RGD)-recognizing integrin receptors, which is mainly expressed in epithelial cells and serves as a binder of ECM endogenous ligands such as fibronectin and latency-associated peptide (LAP) of TGF-β1 and -β3.^14^ In healthy conditions and tissue homeostasis, low or nonexistent expression of integrin αvβ6 is witnessed, apart from specific tissue remodeling conditions involving, for example, hair follicles and epidermis.^14^ The most important physiological function of αvβ6 is the pericellular activation and release of TGF-β1 from the latent TGF-β1 complex triggered by the key interaction between the RGD tripeptide within LAP and the αvβ6 integrin at the αV/β6 interface. The positive feedback loop between TGF-β1 and the αvβ6 integrin ensures mutual surveillance and regulation under physiological conditions. Therefore, dysregulation of the αvβ6/TGF-β1 axis balance with elevated αvβ6 integrin expression and aberrant TGF-β1 activation underlies the etiology of many αvβ6-associated physiological defects and pathologies such as invasive cancer, organ fibrosis, and related inflammation events.^14–16^ While direct targeting of TGF-β1 for fibrosis treatment is not feasible, given its prime role in the regulation of the immune system and general anti-inflammatory surveillance, blocking the localized interaction between αvβ6 integrin and TGF-β1 via suitable antagonist molecules has proven a promising antifibrotic effect. In fact, in recent years, several integrin ligands have been developed and evaluated in either preclinical or advanced clinical trials concerning fibrotic diseases, including the small-molecule bexotegrast (Figure 1B), a dual-selective αvβ6/αvβ1 integrin antagonist,^17,18^ the large-sized antiαvβ6 integrin monoclonal antibody BG00011,^19^ and the nearly 8 kDa-sized αvβ6 or αvβ8-targeted miniproteins recently reported by Baker and Springer.^20^

Also, given the increasing evidence of αvβ6 integrin as a clinically validated fibrosis and cancer target of considerable therapeutic relevance, efforts are being made to conjugate integrin αvβ6 ligands to bioactive moieties to obtain the corresponding dual-active covalent conjugates. The αvβ6 integrin, with its favorable receptor expression profile (i.e., good diseased vs healthy expression ratio), exposed cell-surface location, and ligand-binding-triggered cell internalization, may well enter the shortlist of eligible biomarkers for targeted therapy and companion imaging.^21^ Indeed, αvβ6-directed antibody–drug conjugates (ADCs) have demonstrated useful antitumor activity against multiple αvβ6-positive carcinomas,^22^ and several radioactive αvβ6 integrin-targeted positron emission tomography (PET) tracers have recently proven their diagnostic and prognostic potential in IPF, pancreatic cancer, and primary sclerosing cholangitis.^16,23–26^ Quite surprisingly, the implementation of novel peptide- or small-molecule–drug conjugates (PDCs and SMDCs, respectively), where an integrin αvβ6-directed ligand—either a peptide or an organic small molecule—is linked to a fibrosis-related therapeutic agent, is still lacking.^27^ Indeed, such a conjugate would be highly desirable, en route for targeted delivery of the antifibrotic drug, minimization of the side effects caused by drug therapy alone, and precise inhibition of TGF-β activity within the local microenvironment of the fibrotic lesion.

Along these lines, we have recently reported the design and synthesis of a series of peptide–nintedanib conjugates (PepNINs, Figure 1C) where the αvβ6 integrin-targeted cyclopeptide c­(AmpLRGDL) (with Amp = *cis*-4-amino-l-proline)^28^ was covalently linked to a nintedanib-containing portion through different and chemically robust linkers.^29,30^ In directing their two active “tails” toward the respective αvβ6 integrin and growth factor receptors, dual PepNINs were meant to perturb multiple signaling pathways at a time, resulting in targeted impairment of the profibrotic cascade (Figure 1D,1D′). Recent preliminary *in vitro* studies on the PepNIN series indicated compound **1** (Figure 1C) to be highly promising, since it was able to maintain both the TKI activity against human recombinant VEGFR2 and the binding capability toward αvβ6-positive murine fibroblasts; in addition, it partially and selectively entered αvβ6-positive fibroblasts and exerted *in vitro* antifibrotic properties.^29^

In the present work, our general aim was 2-fold. A first objective was to deeply investigate the *in vitro* and *in vivo* antifibrotic potential of the dual covalent conjugate **1** and to compare its efficacy with that of its individual active components, namely, the αvβ6-targeted cyclopeptide moiety and the cell-untargeted drug nintedanib. To this end, experiments on conjugate **1** were carried out to first assess its chemical stability in murine and human plasma, its affinity toward the isolated αvβ6 integrin receptor, its cell internalization, and antifibrotic activity in human IPF-derived fibroblasts. In addition, molecular modeling studies of the complex between PepNIN **1** and the extracellular portion of the αvβ6 receptor were carried out to evaluate the structural determinants of the ligand–receptor recognition event. Next, the ability of compound **1** to inhibit pulmonary fibrosis progression was evaluated *in vivo* and *ex vivo*, by using a bleomycin (BLM)-induced lung fibrosis mouse model.

A second general objective was to assess *in vivo* and *ex vivo* the role of αvβ6 integrin as well as the disease evolution and localization in a lung fibrosis mouse model. To this end, a novel αvβ6 integrin-targeted conjugate was synthesized, namely, *c*(AmpLRGDL)–ZW800-1 conjugate **2** (Scheme 2), featuring the same RGD-based recognizing unit of the drug-conjugate **1** linked to the known near-infrared (NIR)-active fluorescent probe ZW800-1. Localization of the fluorescent probe **2** in different fibrosis-injured mice lung regions was analyzed together with evaluation of β6 integrin gene expression in fibrotic mice lungs. Overall, the αvβ6-targeting dual conjugates **1** and **2** proved to be competent molecular constructs to be used in αvβ6-associated pathologies such as pulmonary fibrosis therapy and imaging.

## Results

### Synthesis and Plasma Stability of PepNIN **1**

The synthesis of peptide–nintedanib conjugate PepNIN **1** was carried out by following a reported procedure.^29^ Briefly, as detailed in the retrosynthetic Scheme 1, compound **1** could be disconnected along the triazole heterocycle via copper­(I)-catalyzed [3 + 2] cycloaddition, tracing back to the azide-terminating protected cyclopeptide **prot-3** and nintedanib-containing alkyne **4**. Cyclopeptide **prot-3**, as well as its deprotected counterpart **3**, were assembled via Fmoc-based SPPS (solid-phase peptide synthesis) followed by in-solution peptide cyclization.^29^ Alkyne **4**, in turn, was synthesized via the key conjugated nucleophilic substitution between aniline **5** and indolinone enol ether **6**. Of note, the overall chemistry used to construct the nintedanib portion of conjugate **1** (blue portion in formulas of Scheme 1) imitated that used in the original preparation of the nintedanib drug,^31,32^ with the difference that, in our case, an alkynyl PEGylated chain was appended at the piperazine core of aniline **5** instead of a methyl group. Overall, conjugate **1** was obtained on a 10 mg scale as a >99.5% pure compound after reverse-phase high-performance liquid chromatography (RP-HPLC) purification.

**Scheme 1:**
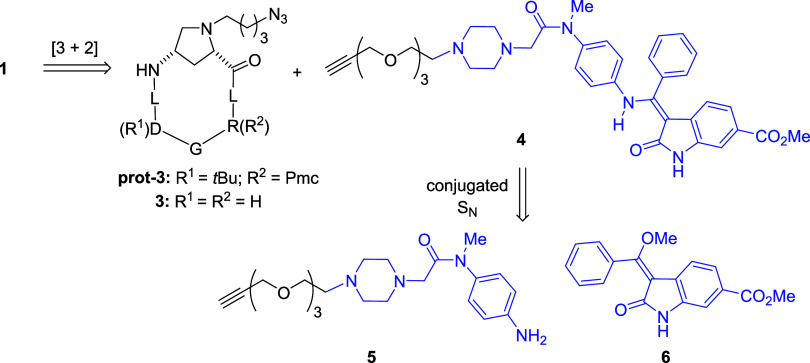
Retrosynthetic Analysis of Peptide–Nintedanib Conjugate **1** ^a^

a The structure of compound **1** is reported in Figure 1C. The blue structural portions are those contained in the nintedanib drug.

The *in vitro* stability of conjugate **1** in mouse and human plasma was evaluated by liquid chromatography–mass spectrometry (LC–MS)/MS analysis, which showed an almost complete resistance to plasma degradation during the observed time (6 h) (see Table S1 in the Supporting Information). This demonstrated that the covalent conjugation of the active “tails” of the molecule resulted in the formation of a robust conjugate designed to survive as a preserved structure during both the *in vitro* and *in vivo* experiments.

### Synthesis and Plasma Stability of Conjugate **2**

As stated in the Introduction, the novel fluorescent conjugate **2** was synthesized, aimed at specifically monitoring the pulmonary fibrotic lesions in our mouse models (*vide infra*). Compound **2** is composed of the αvβ6 integrin-recognizing cyclopeptide **12**—very similar to cyclopeptide **3** embedded in drug conjugate **1**—and the known fluorophore moiety ZW800-1 **10** (Scheme 2). This fluorophore features absorption and emitting wavelengths falling in the NIR-I region (λ_ex_ = 772 nm; λ_em_ = 788 nm), and it previously showed superior properties as compared to other NIR-I emission fluorophores, mainly due to its zwitterionic character.^33^ This structural feature is responsible for enhanced stability, reduced nonspecific binding, and improved overall imaging quality, making it a highly effective *in vivo* diagnostic tool.^33^ Synthesis of **10** followed the footsteps of the originally published reports,^34,35^ with minor modifications involving solvent mixtures and microwave assistance which, in our hands, improved the reactant solubility and overall efficiency. Thus, as shown in Scheme 2, the synthesis started by coupling chlorocyclohexene **7**—obtained by double Vilsmeier–Haack formylation of cyclohexanone followed by bis-imine formation with aniline—with indolium sulfonate **8**, which in turn was prepared from *p*-sulfonated phenyl hydrazine via Fischer indole synthesis followed by *N*-alkylation. The reaction between **7** and **8** was carried out in sodium acetate in ethanol at reflux, which ensured the double Mannich-type addition between the C2-methyl within **8** and the imine groups in **7** followed by aniline elimination. Compound **9** was obtained in 70% yield as a golden green solid, which was used as such in the subsequent step. Chloride **9** was next transformed into phenoxy carboxylate **10** (ZW800-1) by conjugated nucleophilic substitution using the sodium salt of 3-phenoxypropanoic acid in a microwave tube at 65 °C for 45 min. After workup and automated reverse-phase flash chromatography, compound **10** was recovered in 88% yield as a pure, dark green compound. The final assembly of fluorescent conjugate **2** called for amide coupling between the carboxylic acid end of fluorophore **10** and the amine terminus of the peptide moiety. Protected azide-ending cyclopeptide **prot-11**, a protected version of the previously reported αvβ6 integrin binder **11**,^28^ was reduced to amine **prot-12** using zinc and ammonium chloride in methanol/water mixture (79% yield). Finally, amide coupling between amine **prot-12** and carboxylate **10** was carried out using conventional condensation chemistry (hexafluorophosphate azabenzotriazole tetramethyl uranium (HATU), 1-hydroxy-7-azabenzotriazole (HOAt), and diisopropylethylamine (DIPEA) in dimethylformamide (DMF)), providing access to required conjugate **2** after overall acidic deprotection of the cyclopeptide portion (trifluoroacetic acid (TFA), triisopropylsilane (TIS), and H_2_O). Of note, attempts to revert these two final steps, namely, deprotection of **prot-12** to cyclopeptide **12** followed by amide condensation, were not fruitful in our hands, causing degradation of both reactants and products. After purification by automated reverse-phase flash chromatography, compound **2** was recovered as a >98% pure, dark green solid (Figures S1 and S2, Supporting Information).

**Scheme 2:**
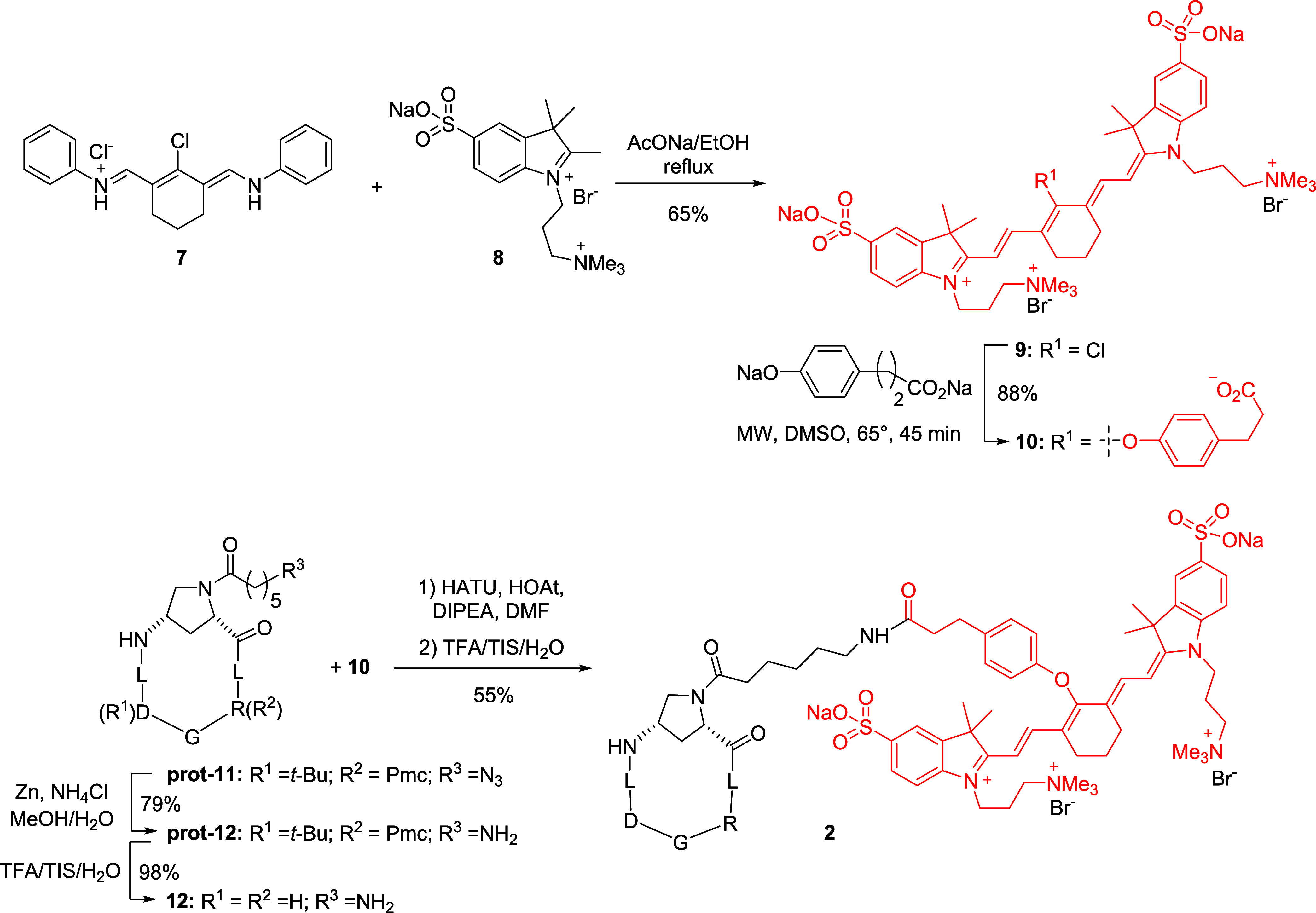
Synthesis of the Novel Peptide–Fluorescent Conjugate **2** ^a^

a The red structural portions are those contained in the fluorescent compound ZW800-1. For details, see the Materials and Methods section and the Supporting Information.

Similar to compound **1**, the *in vitro* stability of conjugate **2** in mouse and human plasma was evaluated by LC–MS/MS analysis, which showed quite good resistance to plasma degradation during the observed time (6 h), with 59 ± 3% recovery of **2** in mouse plasma and 51 ± 3% in human plasma (see Table S1, Supporting Information). These data indicate that fluorescent conjugate **2** is less stable than drug conjugate **1** under the assayed conditions, but it remains sufficiently stable in the time scale of the *in vivo* experiments it was projected for (*vide infra*).

### Evaluation of the Binding Affinity of Conjugate **1** to αvβ6 Integrin

The ability of conjugate **1** to bind to the human, isolated αvβ6 integrin receptor was evaluated by a competitive solid-phase ligand-binding assay on purified αvβ6 integrin in the presence of increasing concentrations (10^–10^–10^–5^ M) of **1**. To better evaluate the impact of the linker and nintedanib moieties on the binding capability of **1**, the results (Table 1) were compared to those obtained for free cyclopeptide **3** and previously reported cyclopeptide **11** (the deprotected version of **prot-11**, Scheme 2). Binding curves are provided in Figure S3, Supporting Information.

**Table 1:** Binding Affinities (IC_50_ Values, nM) of Compounds **1**, **3**, and **11** Determined on Purified αvβ6 Integrin^a^

compound	IC_50_ (nM)^b^ for αvβ6
**1**	1.95 ± 0.16
**3**	4.52 ± 0.68
**11** ^c^	30.3 ± 7.6
*c*(*phg-iso*DGRk)^d^	24.7 ± 3.2

a IC_50_ values were determined by a competitive solid-phase binding assay to LAP.

b Mean ± SD of three independent experiments carried out in triplicate.

c Previously reported data for this compound using a slightly different experimental procedure: IC_50_= 8.3 ± 0.4; SI (IC_50_(αvβ3)/IC_50_(αvβ6)) = 255.^28^

d Used as a reference compound; literature data: IC_50_ = 18 ± 2.5.^36^

As shown in Table 1, conjugate **1** exhibited low-nanomolar affinity toward αvβ6 integrin very similar to that shown by the sole peptide ligand **3**, testifying that the attached linker–drug cargo did not compromise the overall integrin recognition and binding. Moreover, the structurally similar cyclopeptide **11**, previously synthesized^28^ and now used to construct the targeted NIR conjugate **2**, showed comparable binding affinity (Table 1, entry 3) and high αvβ6/αvβ3 selectivity.^28^

### Cell Characterization and Cell Internalization Studies

To evaluate the behavior of conjugate **1** *in vitro*, we considered human fibroblasts from idiopathic pulmonary fibrosis lesions (hIPF fibroblasts). First, we defined whether isolated cells from IPF patients express the biological characteristics of activated myofibroblasts. Cytofluorimetric assays were used to analyze the expression of integrin αvβ6, the target of our ligands and conjugates, as well as the expression of two other antigens namely, CD90 (Thy-1), which is characteristic of mesenchymal-derived cells, and CD326 (EpCAM), which is known to be expressed on epithelial cells. Importantly, integrin αvβ6 was found to be expressed by most of the isolated cells (>97%) (Figure 2A). We also found that approximately 80–85% of the cell population expressed CD90 antigen, while the isolated cell population was negative for CD326 expression, clearly demonstrating that the cells isolated from IPF lesions express a mesenchymal phenotype characteristic of these fibroblasts. Next, we determined the cell internalization of PepNIN **1**, *vis-à-vis* the free nintedanib, via cytofluorimetric analysis, by exploiting the intrinsic fluorescence of the drug moiety (λ_ex_ = 405 nm; λ_em_ = 480 nm), as previously described (Figure 2B).^29^ Cells were exposed for 24 h for treatment with compound **1** or nintedanib at 5 μM concentration; next, the percentage of positive cells of different treatment populations was determined and compared with the fluorescence intensity of untreated (UT) cells. We found that a percentage of about 94% of the cell population was positive when cells were exposed to nintedanib, while a percentage of about 43% of the cell population was positive when the cells were exposed to conjugate compound **1**. Interestingly, the internalization of conjugate **1** was αvβ6 integrin-mediated, as demonstrated by experiments in a previous work.^29^

**Figure 2:**
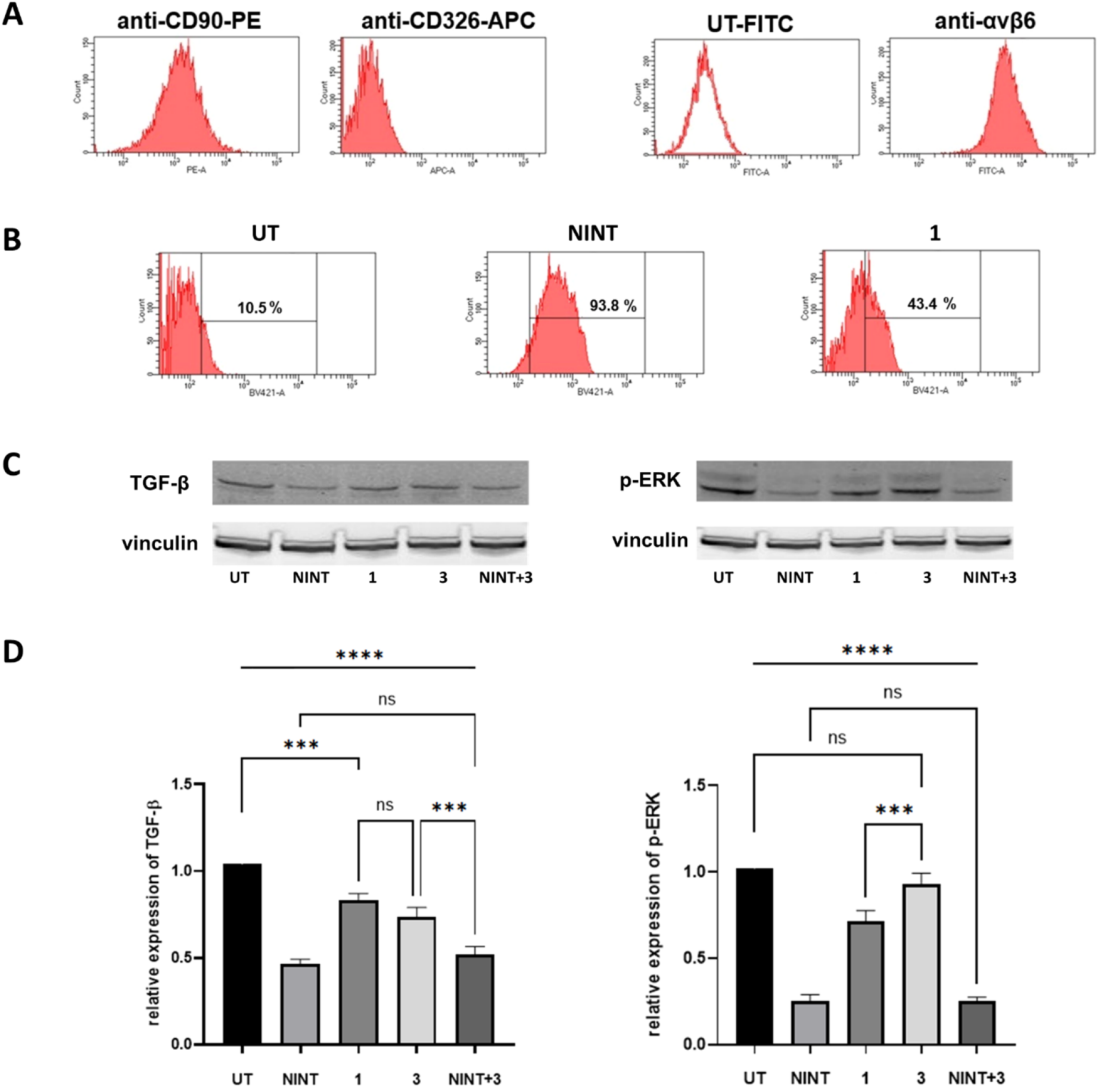
(A) Expression of cell-surface markers of mesenchymal or epithelial differentiation and the αvβ6 integrin receptor in hIPF fibroblasts. Cells were exposed to antihuman CD90-PE, antihuman CD326-APC-conjugated antibodies, or antihuman αvβ6 antibody followed by FITC-conjugated antimouse immunoglobulin (full histograms). As a negative control (open histogram), cells were stained with FITC-conjugated antimouse immunoglobulin alone. (B) Representative images of internalization of conjugate **1** (right) or nintedanib (center), as assessed by flow cytometry measurements. Fluorescence intensity (FacScan FLT1/BV421-A) in hIPF fibroblasts exposed for 24 h to nintedanib or conjugate **1** at 5 μM concentration. The percentages of internalization are reported. As negative control, the autofluorescence of untreated cells was reported (left). (C) Representative Western blot analysis of TGF-β1 (left) and p-ERK1/2 (right) expression in hIPF fibroblasts exposed to nintedanib, compounds **1** and **3**, and to the combination of **3** with nintedanib, for 24 h (5 μM). (D) Densitometric analysis of TGF-β1 (left) and p-ERK1/2 (right) protein expression. Data are representative of three independent experiments, ***p <0.005 and ****p <0.0001. Data are expressed as means ± SEM of fold increment compared to untreated cells normalized to vinculin. Statistical analysis was performed using GraphPad Prism 4 software and posthoc comparison using Tukey’s HSD (honestly significant difference) to determine pairwise differences following the two-way analysis of variance (ANOVA) test.

### Effect of PepNIN **1** and Nintedanib on Human IPF Fibroblasts

The effect of treatment with compound **1** on patient-derived hIPF fibroblasts was next investigated by evaluating the inhibition of both TGF-β1 production and ERK1/2 phosphorylation. It is important to note that TGF-β1 expression is usually evaluated in cultured cells in secreted form in their culture medium (soluble and active form of TGF-β1 (13 kDa)). We reasoned that the evaluation of the intracellular form of full-length TGF-β1 (44 kDa) in hIPF fibroblasts might directly reflect the potency of the conjugated compound to inhibit TGF-β1 production. Evaluation of these fibrosis-related hallmarks would have been indicative of the downstream effect of our conjugate on IPF-related human cell signaling promoted by either the integrin ligand, the nintedanib component, or both.

Thus, we exposed hIPF fibroblasts (passages 4–10, see the Materials and Methods) to 24 h treatment with nintedanib, conjugate **1**, free *c*(AmpLRGDL) cyclopeptide **3**, or the combination of **3** and nintedanib (5 μM, Figure 2C,D). As expected, we found that nintedanib reduced both ERK1/2 phosphorylation and expression of the intracellular form of TGF-β1 (44 kDa). Treatment with compound **1** significantly reduced the ERK1/2 phosphorylation and weakly reduced the latent TGF-β1 production. Free cyclopeptide **3** did not reduce ERK1/2 phosphorylation, and it was able to weakly, yet significantly, reduce the TGF-β1 production, quite similar to the conjugate. Finally, when cells were exposed to the combination nintedanib + cyclopeptide **3**, we obtained a significant reduction of both TGF-β1 and p-ERK1/2, similar to that obtained using nintedanib alone.

These observations would suggest that the component of the nintedanib drug (which exerts its action inside cells) within the conjugate is mainly responsible for the observed behavior, and this could also explain the dependence of the *in vitro* activity of the conjugate upon the extent of its internalized fraction in hIPF cells. In other words, these results confirm the efficacy of nintedanib to inhibit ERK1/2 phosphorylation, which is a key component of the mitogen-activated protein kinase (MAPK) pathway, even in patient-derived IPF cells. The weaker inhibition of ERK1/2 phosphorylation obtained in cells exposed to compound **1** compared to nintedanib may depend on the lower extent of cellular internalization (about halved, Figure 2B). The overall *in vitro* results pointed to a quite inferior performance exerted by the covalent conjugate **1** *vis-à-vis* the free drug in terms of inhibition of downstream signaling connected to integrin αvβ6 and TGF-β. These results also proved inferior as compared to previously reported data of the same conjugate on murine fibroblasts,^29^ possibly due to the intrinsic characteristics of the primary patient-derived IPF fibroblasts in this work.

### Integrin αvβ6 Expression in Lung Fibrosis

**Study #1** aimed to investigate, both *in vivo* and *ex vivo*, the disease evolution and the expression of integrin αvβ6 in a BLM-induced lung fibrosis model. Mice received a triple oropharyngeal administration of saline or BLM, and μCT imaging was carried out on the same animals on 14 and 21 days (Figure 3A).^37,38^

**Figure 3:**
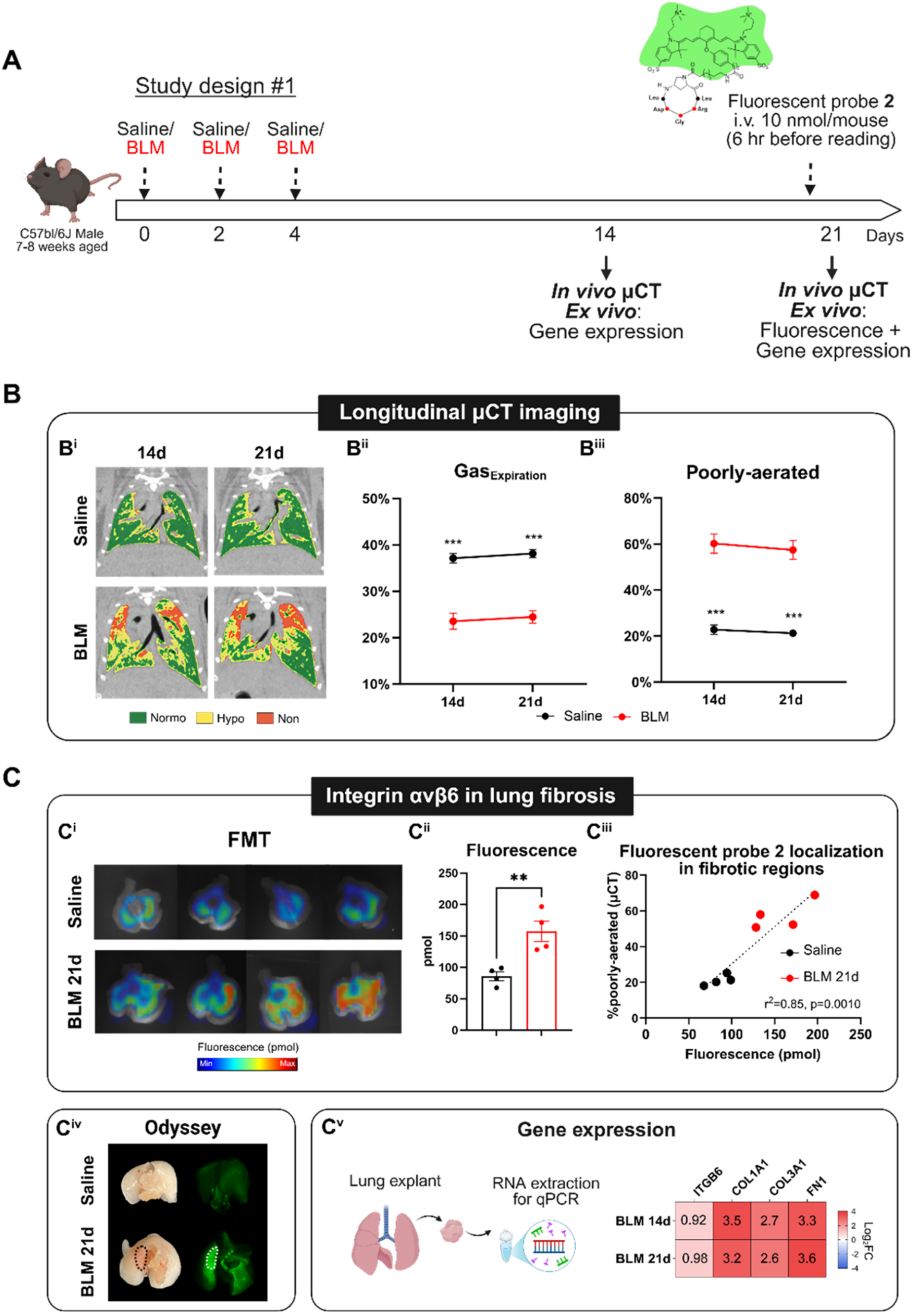
Role of integrin αvβ6 in the lung fibrosis mouse model. (A) Scheme of the experimental setting of **Study #1**. Mice received triple administration of saline or BLM and were longitudinally imaged by μCT on days 14 and 21 to monitor lung fibrosis development. (B) Some representative coronal lung images with segmentation masks of aeration compartments, e.g., normo-, hypo-, and nonaerated (B^i^), %Gas_Expiration_ (B^ii^), and poorly aerated areas (B^iii^) longitudinal quantification from each computed tomography (CT) scan in both Saline and BLM groups. (C) On day 21, a subset of mice (*n* = 4 Saline; *n* = 4 BLM) were injected with 10 nmol/mouse fluorescent conjugate **2**, and after 6 h lungs were explanted for fluorescence quantification by fluorescence molecular tomography (FMT) (C^i,ii^), and results were then correlated with the poorly aerated parameter derived by μCT (C^iii^). Lungs were also scanned with an Odyssey (LI-COR) to better visualize the fluorescence probe localization in fibrotic areas (C^iv^). Gene expression analysis on lung tissues was conducted for all mice on days 14 and 21 (*n* = Saline; *n* = 4 BLM at each time point); Log_2_(fold change) of Itgb6, Col1a1, Col3a1, and Fn1 genes were reported for BLM 14d and BLM 21d groups compared to Saline (C^v^). Data were expressed as mean ± SEM. Two-way ANOVA with Dunnett’s test for multiple comparisons were applied for CT-derived parameters, ***p <0.001 vs BLM group. Parametric *t* test was utilized to compare fluorescence signals between Saline and BLM groups, **p <0.01. Pearson coefficient (*r* ^2^) and *p*-value were measured to evaluate the relationship between fluorescence signals and fibrosis assessment by μCT.

Representative coronal CT images from saline and BLM-treated mice at both 14 and 21 days are shown in Figure 3B^i^. The colored masks represent different lung aeration degrees, where *green* areas represent the normally aerated tissue, whereas *yellow* and *red* portions correspond to moderate-to-severe fibrotic regions (globally named poorly aerated areas). As expected, longitudinal μCT revealed a significant decline in %Gas_Expiration_ and an increase in poorly aerated regions in BLM-treated animals compared to saline at both time points (*p* <0.001), confirming persistent lung fibrosis lesions mainly localized in the apical part.^37,39–41^

To investigate the expression of integrin αvβ6 *ex vivo*, a subset of saline and BLM mice were intravenously injected with fluorescent conjugate **2** at 21 days, and then 6 h later they were euthanized for lung fluorescence quantification. Fluorescence molecular tomography (FMT) was used to detect the fluorescence signal resulting from the specific binding of compound **2** to its target, integrin αvβ6.^42,43^ Although the images presented in Figure 3C^i^ are for illustrative purposes, a clear visual distinction between the significant signals between fibrotic and healthy subjects is readily apparent. Furthermore, the fluorescence quantification corroborated these findings, with a statistically significant difference between the two groups (*p* <0.01, Figure 3C^ii^). The strong association between the fluorescence signal and poorly aerated lung regions measured by μCT (*r* ^2^ = 0.85, *p* = 0.001) suggested that integrin αvβ6 may be primarily expressed in moderate-to-severe fibrosis (Figure 3C^iii^). To further support this hypothesis, saline and BLM lungs were also scanned using Odyssey DLx, displaying fluorescence localization in the apical region, which is more severely affected by fibrosis (Figure 3C^iv^). Gene expression analysis on lung tissues revealed that several genes, including Itgb6 (integrin β6), Col1a1, Col3a1, and Fn1 (components of the extracellular matrix), were upregulated in BLM samples at both 14 and 21 days compared with healthy controls (Figure 3C^v^).

Overall, these data demonstrated that integrin αvβ6 is significantly expressed during lung fibrosis development, highlighting its specific role in the BLM model, and that fluorescent probe **2** is functional to specific visualization of the affected fibrotic regions of mice lungs.

### *In Vivo* Antifibrotic Therapy Assessment

Figure 4A illustrates the setup of the pharmacological experiment identified as **Study #2** (see also Table S2 in the Supporting Information). After triple BLM instillation, all mice underwent μCT imaging on days 14 and 21, indicating the beginning and the end of the therapeutic window.^37,39^ On day 14, the BLM-treated mice were divided into 5 groups, namely, Vehicle, NINT (23), NINT (94), PepNIN **1** (23), and Ligand **11** (23). The reference standard in our drug discovery studies is NINT (94), representing the ideal dosage (94 μmol/kg) of the FDA-approved drug that produces antifibrotic effects in the preclinical BLM model.^37^ To better emphasize the synergistic therapeutic effect of nintedanib when it is covalently conjugated with our integrin ligand, the stoichiometric dose of nintedanib within the PepNIN **1** group was reduced by four times (to 23 μmol/kg). The two remaining groups received the NINT (23) (nintedanib at 23 μmol/kg) and ligand **11** (23) (cyclopeptide ligand **11** at 23 μmol/kg) alone to investigate the therapeutic effect of each single component at this reduced dosage. As expected, BLM administration caused a 10–15% of body weight reduction within the first 7 days compared to the saline group. All treatments were well tolerated with no signs of animal distress or significant differences in body weight loss within groups. From 14 to 21 days, PepNIN **1** (23) and NINT (94) partially gained weight loss compared to integrin ligand **11** (23) and NINT (23) (Figure S4 in the Supporting Information).

**Figure 4:**
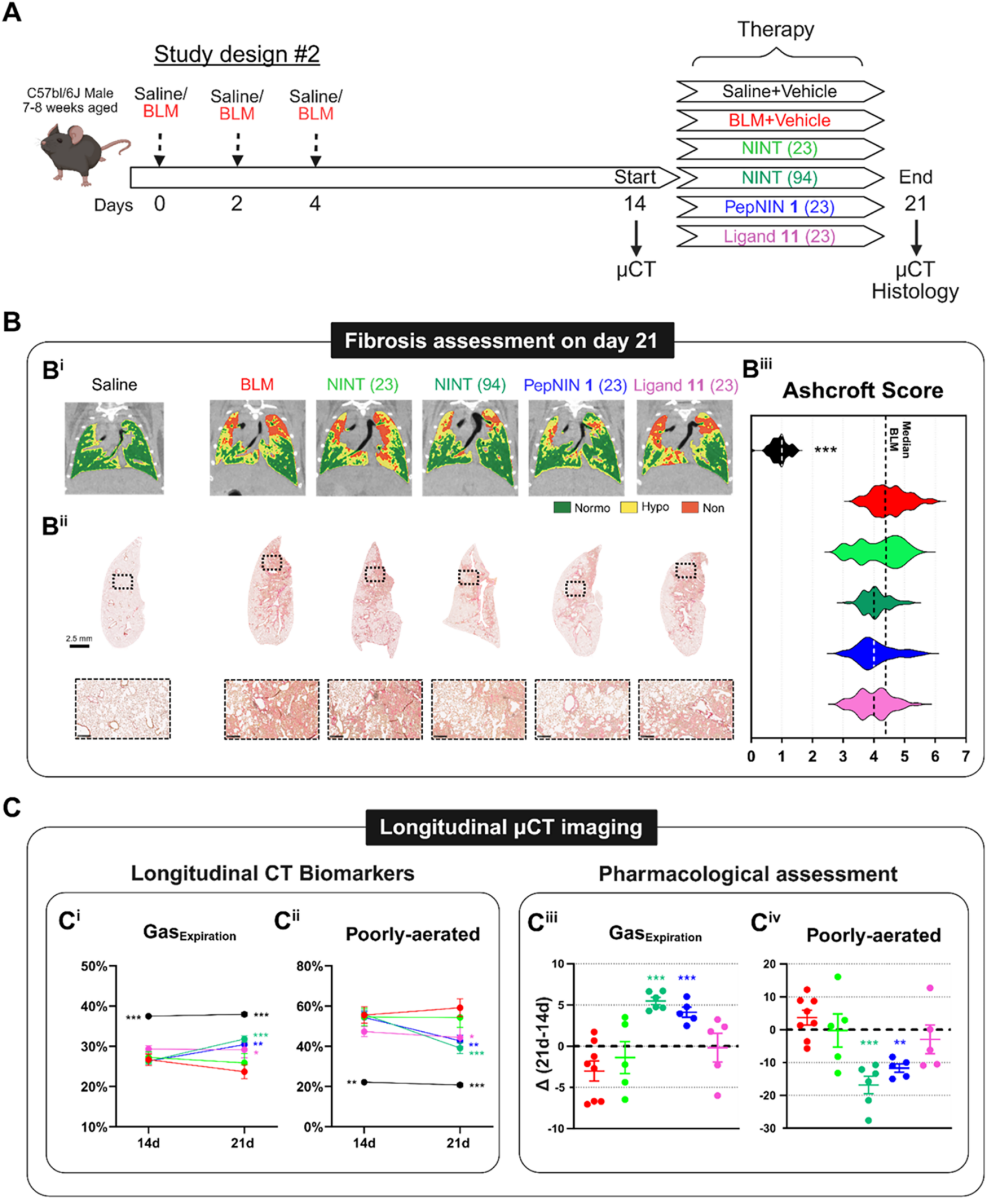
Antifibrotic effects of nintedanib, PepNIN **1**, and cyclopeptide **11** in pulmonary fibrosis. (A) Scheme of the experimental setting of **Study #2**. Mice received triple administration of Saline or BLM. On day 14, BLM-treated mice were divided into 5 groups and orally treated daily with vehicle, NINT, PepNIN **1**, and **11** at 23 μmol/kg or NINT at 94 μmol/kg up to day 21. All mice were longitudinally imaged by μCT on days 14 and 21 to monitor lung fibrosis development and were culled at the end point for the histological analysis. (B) Representative coronal CT images (B^i^) and the corresponding histological slides of the left lobe stained with Picrosirius red on day 21 (B^ii^). Ashcroft score (AS) quantification of each group is presented as a violin plot displaying different fibrosis severities (B^iii^). (C) For each group, CT-derived biomarkers (e.g., %Gas_Expiration_ and poorly aerated tissue) were measured longitudinally (C^i,ii^) and adjusted to their basal value on day 14 (start of treatment) (C^iii,iv^). Since the saline group did not display any variations over time, it was reported as a dotted line at *y* = 0 (C^iii,iv^). Data were expressed as mean ± SEM. Two-way ANOVA with Dunnett’s test for multiple comparisons was used to compare longitudinal CT-derived parameters at each time point, *p <0.05, **p <0.01, ***p <0.001 vs BLM group. One-way ANOVA with Dunnett’s test for multiple comparison was used to compare variations in CT-derived parameters, **p <0.01, ***p <0.001 vs BLM group.

Typical coronal CT images highlighting morphological lung parenchymal abnormalities for each group at the end of the study are reported in Figure 4B^i^, suggesting that NINT (94) and PepNIN **1** (23) reduced the extent of fibrotic lesions corresponding to hypo- and nonaerated areas compared to BLM animals. Histological staining with Picrosirius red (Figure 4B^ii^) and Ashcroft score quantification (Figure 4B^iii^) corroborated μCT observations.^42,43^ The BLM group showed a significant increase in collagen deposition mainly localized in the peribronchial areas in the apical lobe, as well as the number of fields with moderate-to-severe fibrosis (Ashcroft score ≥4) compared to Saline, which were characterized by normal tissues. None of the treatments was able to substantially decrease collagen deposition when compared to BLM; nonetheless, NINT (94) and PepNIN **1** (23) clearly reduced severe fibrotic lesions, as shown in violin plots. Cyclopeptide **11** (23) only partially affected fibrosis, whereas NINT (23) had no effects (Figure 4B^iii^).

Longitudinal μCT aeration biomarkers^37,39^ revealed that all BLM-treated animals had comparable lung fibrosis degree prior to start of the treatment, as shown by percentages of Gas_Expiration_ and poorly aerated tissue (Figure 4C^i,ii^). From day 14 to day 21, the BLM-treated animals exhibited a tendency toward disease progression,^37^ and a similar trend was also observed in mice receiving NINT (23). In contrast, NINT (94) and PepNIN **1** (23) significantly improved lung aeration compared to the BLM group, while integrin ligand **11** (23) only stabilized the pathology development (Figure 4C^i,ii^).

In order to accurately estimate the onset of the disease, to better assess the efficacy of the treatment, and to minimize intra-animal variation, the CT parameters of each mouse were adjusted by its own basal value on day 14. This visualization showed a similar disease progression in the BLM, NINT (23), and ligand **11** (23) groups. However, mice treated with NINT (94) and PepNIN **1** (23) showed statistically significant improvement on both CT biomarkers (*p* <0.001 for gas expiration; *p* <0.01 and *p* <0.001 for poorly aerated regions, respectively, Figure 4C^iii,iv^).

### Structural Modeling of the αvβ6 Integrin–PepNIN **1** Complex

A docking approach to model the interaction of RGD peptide–nintedanib conjugate PepNIN **1** with integrin αvβ6 was developed. Docking calculations were carried out with the Glide software package starting from the X-ray structure of the extracellular segment of integrin αvβ6 in complex with a RGD-containing peptide from the TGF-β3 prodomain (PDB code: 4UM9),^44^ according to the procedure reported in the Materials and Methods. As observed in other X-ray structures of integrins in complex with RGD ligands,^45–48^ the RGD sequence in an extended conformation binds at the interface of the α and β subunits with the carboxylic and guanidine groups acting as an electrostatic clamp, respectively, on a bivalent cation of the β subunit (MIDAS, metal-ion-dependent adhesion site) and on specific acid residues of the α subunit (e.g., Asp218 in the αv subunit).

In order to properly accommodate the peptide–nintedanib conjugate, a new model of the αvβ6 integrin was generated by setting a larger receptor grid (the largest possible size of 48 Å outer box was used), compared to the models employed in previous docking calculations of cyclic RGD peptidomimetics into the αvβ6 binding site.^28,49^ A rigid receptor-flexible ligand docking protocol was set up and then applied to predict the mode of binding of the conjugate to αvβ6 integrin by adopting an incremental growth strategy for the ligand. Indeed, the latter was built and docked into the binding site in several successive steps by gradually increasing its size, according to the fragmentation shown in Figure S5 in the Supporting Information.

The first structure used in docking calculations contains the cyclic RGD peptide functionalized at proline nitrogen with a handle ending with the triazole and the PEGylated linker (fragment 1 in Figure S5). As previously observed in docking studies of Amp-based αvβ6-selective cyclopeptidomimetics,^28^ the ligand is accommodated into the αvβ6 binding site, displaying an RGD extended conformation that allows establishing the key polar/electrostatic interactions observed in the X-ray structure, i.e., the bidentate side-on interaction of the Arg guanidinium group with the side chain of αv-Asp218, and the coordination of the Asp carboxylate to the Mg^2+^ ion in the MIDAS region. Moreover, the oxygen atom of the RGD aspartate side chain not engaged by MIDAS Mg^2+^ ion forms hydrogen bonds with backbone NH groups of β6-Ala126 and β6-Asn218. Other stabilizing hydrogen-bond interactions occur between the backbone Gly carbonyl and Asp-NH ligand moieties and the β6-Thr221 side chain and β6-Ile219 carbonyl group, respectively (Figure 5). Moreover, the Leu residue flanking the Asp of the RGD motif points to the residue Ile183, a hot-spot residue of the β6-specific hydrophobic pocket close to the RGD binding site. The flexible proline exocyclic appendage is mostly oriented toward the adjacent-to-MIDAS metal ion (ADMIDAS) and its coordinating residues, adopting different conformations and contacting various receptor residues in the β6 subunit (e.g., Lys338 in the top-ranked poses).

**Figure 5:**
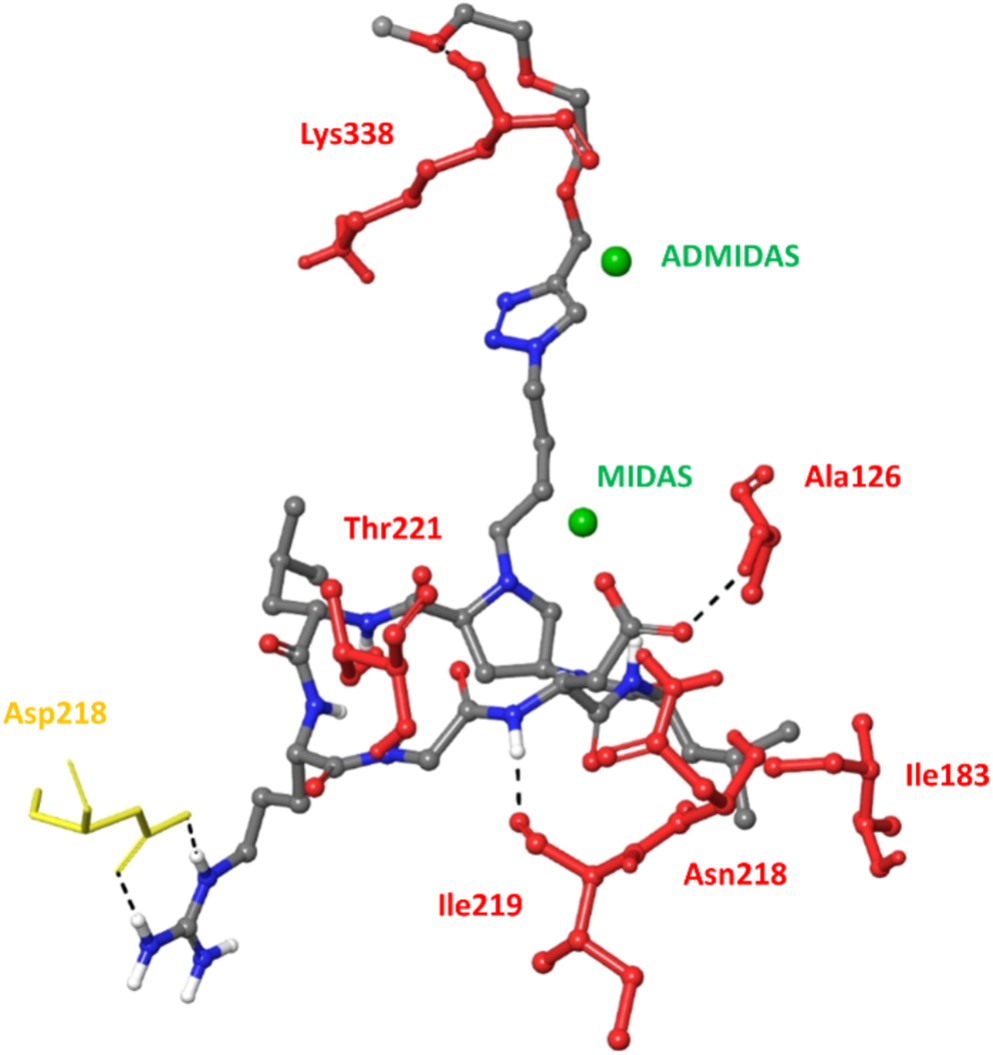
Docking best poses of fragment 1 from conjugate **1** into integrin αvβ6 (4UM9 PDB). Only selected integrin residues involved in interactions with the ligand (gray sticks) are shown and labeled in yellow for αv and red for β6. Nonpolar hydrogens are hidden for clarity; intermolecular hydrogen bonds are shown as black dashed lines.

Starting from the best pose achieved from the docking of the first fragment, the molecule size was incremented by adding the piperazine fragment, and the resulting structure (fragment 1 + fragment 2, Figure S5 in the Supporting Information) was docked using the same protocol. The cycle was repeated by including the aniline fragment (fragment 3), and finally the complete peptide–nintedanib conjugate PepNIN **1** was generated from different orientations of the flexible chain obtained in the previous step. Docking results show that the macrocycle portion is stable within the pocket and superimposable binding modes, maintaining the key interactions of the RGD motif, and can be identified among calculated docking poses for all of the intermediate structures and the entire conjugate. On the contrary, high variability is observed for the position of the flexible appendage bearing the drug in the conjugate, which is shown to sample several conformations and contact different regions in the integrin β6 subunit, overall exploring a rather large area (Figure 6), ranging from the ADMIDAS region and the hydrophobic pocket to the interface with the αv subunit. In conclusion, a multistep docking protocol was applied to generate 3D computational models for interaction of the RGD conjugate with αvβ6 integrin and evaluate its ability to properly fit the receptor site. In agreement with the experimental binding data, the large functionalization conjugated to the Amp residue in the cyclopeptide does not affect the interaction of the RGD recognition sequence, thus allowing the ligand to fit unhindered the binding site.

**Figure 6:**
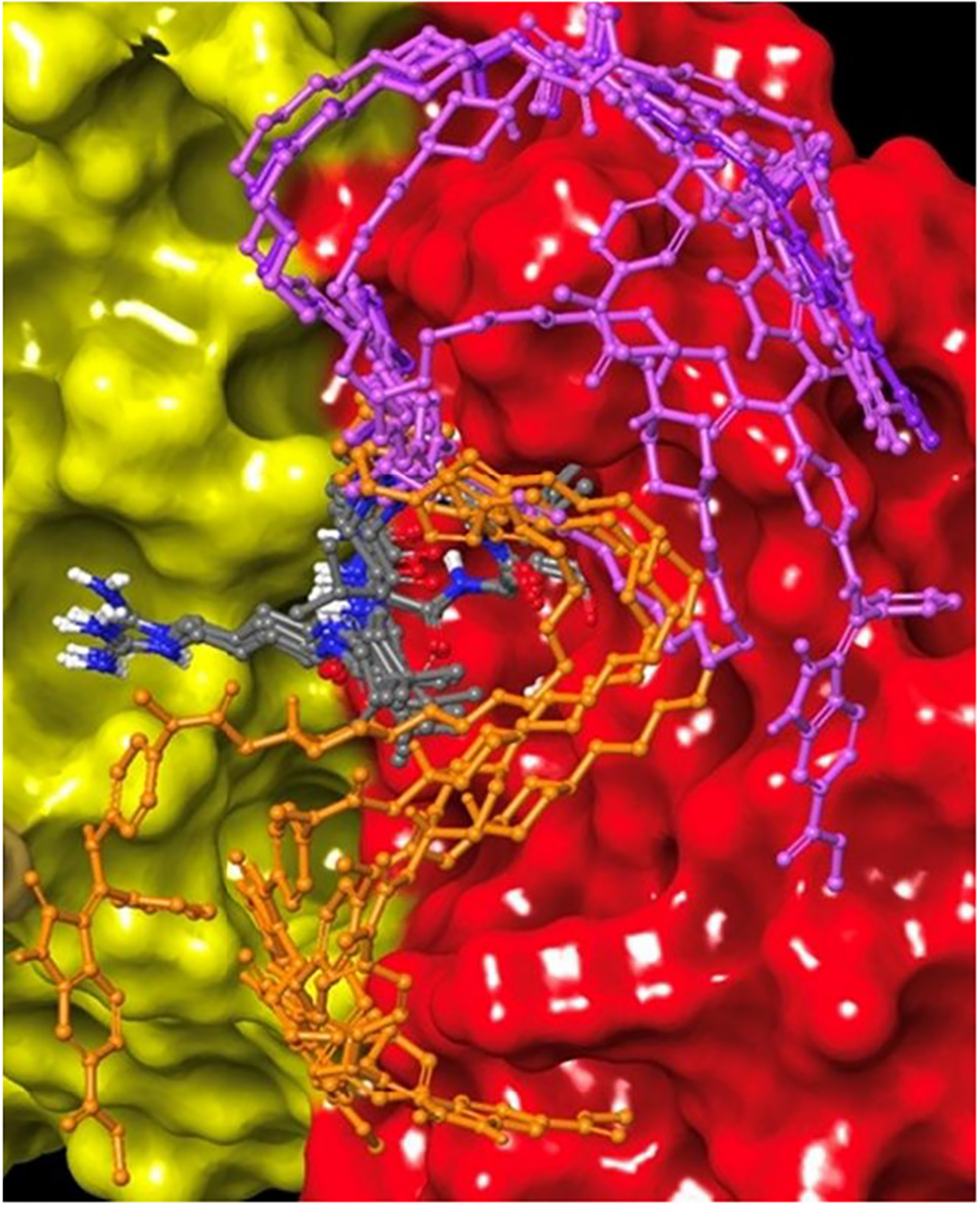
Calculated docking poses for the RGD peptide–nintedanib conjugate PepNIN **1** to form the αvβ6 binding site. The conjugate is reported as gray sticks for the cyclopeptide portion and as orange or violet sticks for the linker–drug portion. The αvβ6 receptor is depicted in yellow (αv subunit) and red (β6 subunit) surfaces.

## Discussion

The limited efficacy of current approved antifibrotics and their poor tolerability among a notable proportion of patients have renewed interest in developing targeted therapeutics for IPF.^4,5,11^ Single-agent αvβ6 integrin-targeted compounds have been developed in recent years, with varied outcomes. For example, the large-sized antiαvβ6 integrin monoclonal antibody BG00011, after promising early-stage clinical phase results, was prematurely terminated due to adverse pro-inflammatory behavior, possibly given by the differential penetration within the dense fibrotic matrix vs nonfibrotic regions of the lung.^19,50^ On the other hand, the dual αvβ6/αvβ1 small-molecule bexotegrast (Figure 1B) entered the late-stage Phase 2b clinical IPF trial, which was very recently discontinued for safety reasons, while showing strong evidence of efficacy.^17,18,51^ However, considering that fibrogenesis is complex, dynamic, and multifactorial, an antifibrotic approach addressing multiple key targets simultaneously in the local microenvironment of the fibrotic lesion could be a highly promising option.

The <2 kDa-sized compound PepNIN **1** developed in this work is made by the covalent connection of a αvβ6 integrin-targeted AmpRGD-based cyclopeptide to multi-GFR-directed therapeutic drug nintedanib via a robust triazole-PEG linker (Figure 1C). By its inherently composite chemical nature, this dual conjugate has been specifically designed to inhibit the multiple and intertwined fibrosis-related TGF-β1/αvβ6 integrin/GFR axis. In addition, the simple and modular synthesis of **1** suggested the preparation of the novel fluorescent αvβ6-directed companion probe **2**, to overall provide a precious therapeutic and imaging active molecular couple.

Crucial issues are included in the design of conjugates **1** and **2**. *First*, the two peptide and drug-active modules of **1** should maintain their comparable low-nanomolar affinity toward the respective integrin and GF receptors, and the expected therapeutic action could be given by the joint contribution of both. In most covalent conjugates, instead, the overall activity is mainly dictated by the highly cytotoxic drug payload, relegating the ligand to the major role of the directing unit. *Second*, given the robustness of the designed linkers in **1** and **2**, they are meant to survive as intact constructs during the experimental timelapse, with minimal (if any) premature leakage of the constituting modules. *Third*, for the theranostic couple of conjugates **1** and **2** to be effective, active (at least partial) internalization in αvβ6-overexpressing cells is required.

The *in vitro* and *in vivo* experiments presented in this study corroborated the previously reported initial results^29^ and widely demonstrated the therapeutic and fluorescence imaging potential of our model compounds **1** and **2**. The synthesis and purification of **1** and **2** were quite simple and reliable (Schemes 1 and 2); furthermore, these compounds proved to be quite resistant to both mouse and human plasma within 6 h (Table S1 in the Supporting Information), in line with our initial requirements. Interestingly, low-nanomolar-binding affinity of conjugate **1** toward the isolated αvβ6 receptor was witnessed, in line with the binding capability of the free AmpRGD peptides **3** and **11** (Table 1). Again, this result agreed with the design requirements, that is, the two active components do not interfere with each other, yet they maintain their native low-nanomolar recognition capability toward the respective targets.^52^ The rationale for this behavior was provided by molecular modeling studies of **1** in complex with the integrin αvβ6. This study revealed the maintenance of key interactions as well as perfect alignment of the AmpRGD cyclopeptide unit within the αvβ6 recognition pocket while leaving the appended linker–drug moiety “fluctuating” along the other regions of the receptor (Figures 5 and 6).

Using primary fibroblasts from IPF patients, which proved to overexpress the αvβ6 integrin, cell uptake studies and evaluation of the impact of **1** on TGF-β-related downstream signaling were carried out, *vis-à-vis* the free drug and peptide ligand counterparts. By cytofluorimetric analysis, it was demonstrated that PepNIN **1** partially entered these cells in about a halved percentage with respect to the free drug (43.4 vs 93.8%, Figure 2B). Noticeably, the αvβ6 integrin-mediated internalization of **1**, as well as the nonspecific uptake of nintedanib were demonstrated using αvβ6-positive and αvβ6-negative cells in a previous work.^29^ Compound **1** weakly, yet significantly, reduced both the intracellular TGF-β1 production and ERK1/2 phosphorylation with comparable or superior ability as compared to free ligand **3**, but with inferior performance with respect to free nintedanib or the combination nintedanib + ligand **3**. This behavior could be due to the reduced cell uptake of **1** as compared to the free nintedanib; indeed, the actual added value of the targeted dual conjugate **1** compared to the single components could be fully revealed just via *in vivo* experiments.

In a first *in vivo* study (**Study #1**), a combination of different methods comprising *in vivo* μCT imaging, *ex vivo* lung FMT and Odyssey scanning, as well as *ex vivo* gene expression analysis of integrin β6 was carried out on a BLM-induced murine lung fibrosis model, according to the experimental setting outlined in Figure 3A and using the targeted compound **2** as a key fluorescent probe. The results reported in Figure 3 collectively showed the following: (i) specific uptake of the targeting probe in the diseased tissues with evidence of αvβ6 integrin overexpression and mapping and (ii) direct correlation between fluorescent probe/αvβ6 localization and the severity degree of disease in diversely affected regions of the lungs. In other words, a sort of targeting effect within the lungs was observed using the fluorescent conjugate **2**.

Then, the antifibrotic efficacy of PepNIN **1** in BLM-treated mice models was demonstrated in comparison to the free nintedanib and AmpRGD components (**Study #2**). Importantly, the dosage of conjugate **1** was 4-fold reduced at 23 μmol/kg with respect to the standard dose (94 μmol/kg) usually used for the free drug in these BLM-challenged mice models, to better emphasize the likely improved therapeutic effect of the covalent presentation as compared to the single active units. Overall, *in vivo* μCT and *ex vivo* histology pointed to the following results: the extent and severity of diseased lung areas and fibrotic lesions in BLM-treated mice were clearly and significantly reduced upon treatment of both NINT (94) and PepNIN (23) (Figure 4). Importantly, the antifibrotic efficacy of the conjugate PepNIN **1** at 23 μmol/kg (blue color in Figure 4) closely compared that of nintedanib at 94 μmol/kg (dark green), while the free components nintedanib (light green) and cyclopeptide **11** (pink) at reduced 23 μmol/kg dosage were only weakly effective, supporting the initial key hypothesis of this work, namely, that the covalent presentation is superior in efficacy with respect to the single components. Finally, measurements of μCT biomarkers (%Gas_Expiration_ and % of poorly aerated areas) in the 14d–21d therapy timelapse and intra-animal variation adjustment confirmed a marked, comparable, and statistically significant antifibrotic outcome upon NINT (94) and PepNIN **1** (23) treatment, disease progression with NINT (23) and BLM, and almost steady conditions with ligand **11** (23) (Figure 4C). Finally, oral daily administration of all treatments was well tolerated, with no sign of significant body weight loss (Figure S4 in the Supporting Information).

## Conclusions

In this study, we developed a couple of molecular dual conjugates, namely, the peptide–nintedanib conjugate PepNIN **1** and the peptide–NIR probe **2**, which served as useful αvβ6 integrin-targeting therapeutic/imaging tools in lung fibrosis mouse models. Collectively, the exquisite binding capability toward the αvβ6 integrin, good resistance to plasma degradation, facile synthetic access, and ability to partially enter αvβ6-overexpressing IPF patient-derived cells of either or both of these conjugates constituted good premises for further exploration of their potential *in vivo*. In fact, in BLM-induced lung fibrosis mouse models, significant correlation was shown between the expression of the αvβ6 integrin during the disease progression and the detected fluorescence of probe **2**, as well as its specific localization in the most severely injured regions of the lungs, supporting the potential usefulness of conjugate **2** as a noninvasive diagnostic agent of this pulmonary fibrosis model. The nintedanib conjugate **1**, on the other hand, proved its antifibrotic efficacy in the same pulmonary fibrosis model, and it demonstrated its superior therapeutic effect with respect to the separated active modules without impacting mice vitality.

Given the wide therapeutic application of the nintedanib drug and the involvement of dysregulation of the TFGβ-αvβ6-GFR axis in the etiology of other pathologies including other organ fibrosis and diverse solid tumors, we anticipate that this targeted molecular couple (or structurally modified analogues) could serve as a proof-of-principle tool beyond the lung fibrosis field and embrace other diseases.

## Materials and Methods

### Chemistry

#### Synthesis of Conjugate **2**

In a round-bottomed flask equipped with a magnetic stir bar and kept under a nitrogen atmosphere, compound **10** (9 mg, 6.6 μmol, 1.5 equiv) was dissolved in DMF (300 μL), and then HATU (3.37 mg, 0.0089 mmol, 2 equiv) and DIPEA (9 μL, 0.0517 mmol, 11 equiv) were sequentially added. The reaction was left stirring for 5 min, and then **prot-12** (5.9 mg, 4.4 μmol, 1 equiv) was added. The reaction was left stirring for 2 h, and then the reaction mixture was evaporated. The resulting crude mixture was purified by automated flash chromatography using the following solvent system: H_2_O + 0.1% TFA (solvent A) and ACN (solvent B); λ_nm_ detected: 220/700 nm; method: from 20% to 100% solvent B. A bright-green solid was recovered and was treated with a solution of TFA/TIS/H_2_O (95:2.5:2.5). The reaction was left stirring for 1.5 h; then the solvent was removed, and the crude was purified with automated flash chromatography using the following solvent system: H_2_O + 0.1% TFA (solvent A) and ACN (solvent B); λ_nm_ detected: 220/700 nm; method: from 20 to 100% solvent B. Product **2** was obtained as a bright-green solid (4.7 mg, 55% two-step yield from **10**). ^1^H NMR (400 MHz, CD_3_OD) δ 8.08 (d, *J* = 13.8 Hz, 2H), 7.94–7.76 (m, 4H), 7.37 (dd, *J* = 8.7, 3.0 Hz, 2H), 7.28 (d, *J* = 8.4 Hz, 2H), 7.05 (dd, *J* = 8.5, 3.6 Hz, 2H), 6.28 (d, *J* = 14.9 Hz, 2H), 4.53–4.29 (m, 4H), 4.22 (m, 5H), 4.14–4.06 (m, 1H), 4.07 (d, *J* = 17.5 Hz, 1H), 3.94–3.84 (m, 1H), 3.81 (d, *J* = 17.5 Hz, 1H), 3.65–3.53 (m, 4H), 3.43–3.32 (m, 1H), 3.29–3.23 (m, 2H), 3.20 (s, 18H), 3.18–3.12 (m, 2H), 3.12–3.05 (m, 2H), 3.06–2.97 (m, 1H), 2.95–2.77 (m, 8H), 2.59–2.49 (m, 1H), 2.48–2.37 (m, 2H), 2.38–2.22 (m, 4H), 2.19–2.01 (m, 2H), 1.99–1.84 (m, 4H), 1.81–1.58 (m, 10H), 1.58–1.48 (m, 2H), 1.39 (s, 12H), 1.02–0.83 (m, 12H). HRMS (ES+) C_86_H_126_N_15_O_17_S_2_^+^ calcd for [M + 3H]^3+^ 569.3042; found 569.3024 [M + 3H]^3+^. Purity, >98%. The HPLC trace and HRMS spectrum are shown in Figures S1 and S2 in the Supporting Information.

### *In Vitro* Biological Studies

#### Solid-Phase αvβ6 Integrin Binding Assay

The affinity of integrin ligands **1**, **3**, and **11** was determined by the solid-phase binding assay using soluble α_v_β_6_ integrin and coated LAP, according to a previously reported protocol.^36^ Flat-bottom 96-well plates (Corning Costar, Segrate, Milan, Italy) were coated by passive adsorption with LAP (TGF-β; 0.4 μg/mL; R&D Systems) in carbonate buffer (15 mM Na_2_CO_3_ and 35 mM NaHCO_3_, pH 9.6; 100 μL/well) overnight at 4 °C. After three washes with PBS-T buffer (phosphate-buffered saline–Tween20, 137 mM NaCl, 2.7 mM KCl, 10 mM Na_2_HPO_4_, 2 mM KH_2_PO_4_, and 0.01% Tween20, pH 7.4; 200 μL/well), wells were blocked with TS-B buffer (Tris–saline/BSA buffer; 20 mM Tris–HCl, 150 mM NaCl, 1 mM CaCl_2_, 1 mM MgCl_2_, 1 mM MnCl_2_, pH 7.5, and 1% BSA; 150 μL/well) for 1 h at room temperature. After washing the assay plate three times with PBS-T (200 μL/well), soluble human α_v_β_6_ integrin (0.25 μg/mL, R&D Systems) was incubated with compounds and tested at different concentrations (10^–5^–10^–10^ M), in the coated wells for 1 h at room temperature. The plate was washed three times with PBS-T buffer, and anti-α_v_ primary antibody (1:500 dilution, 100 μL/well; mouse antihuman MAB1978, Merck Life Science, Milan, Italy) was added and incubated for 1 h at room temperature. Then, the secondary peroxidase-labeled antibody (2.0 μg/mL, 100 μL/well; HRP-conjugated goat antimouse IgG (H + L), Proteintech, DBA, Segrate, Milan, Italy) was added to the plate after three washes with PBS-T and incubated for 1 h at room temperature. After washing the plate three times with PBS-T, the plate was developed by quick addition of 50 μL/well of TMB (3,3′,5,5′-tetramethylbenzidine liquid substrate system, Merck Life Science) and incubated for at least 4 min at room temperature in the dark. The reaction was stopped with 1 M H_2_SO_4_ (50 μL/well), and the absorbance was measured at 450 nm with an EnSpire Multimode Plate Reader (PerkinElmer, Waltham, MA). Experiments were carried out in triplicate and repeated at least three times. Data analysis and IC_50_ affinity values were calculated using GraphPad Prism 10.4.1 (GraphPad Software, San Diego, CA). Solid-phase binding curves are shown in Figure S3 in the Supporting Information.

### Cell Cultures

#### Ethics

IPF cells were isolated from patients who underwent surgical resection of lung cancer with concomitant idiopathic pulmonary fibrosis. The University of Florence and Azienda Ospedaliero Universitaria Acreage Board approved the study (CT01 26703/oss/2024 Territorial Ethics Committees CET). All of the samples were obtained through the informed consent of the donors involved in the study. The informed consent was drafted in accordance with the EU General Data Protection Regulation (GDPR) 2016/679 and with the Italian Legislative Decree No. 211/2003, which sets out provisions for experimental studies performed using biological samples.

IPF tissue specimens were obtained distant from the lung cancer lesion. Tissue specimens were minced using a sterile straight scissor on ice into tiny pieces smaller than 1 × 1 mm^2^ within 10–15 min. Specimens were allowed to grow in plastic dishes under glass coverslips and maintained in a controlled humidified atmosphere at 37 °C with 5% CO_2_. Cultures were maintained in FBMTM medium (CC-3131 FBM Fibroblast Growth Basal Medium, Lonza) with BulletKit (CC-4126, Lonza) with the following components: insulin (0.5 mL/500 mL), rhFGF-B (r-human fibroblast growth factor-B, 0.5 mL), GA-100 (gentamicin sulfate and amphotericin B, 0.5 mL), 10% fetal bovine serum (FBS, Gibco) and 1% penicillin–streptomycin (EuroClone, Milan, Italy). After 1 week, emerging cells from the samples were subcultured and amplified (passage 0). Subconfluent cultures were detached by incubating with 1× trypsin–EDTA solution (EuroClone, Milan), and subcultured every 3 days at a ratio of 1:3. Cells at passages 4–10 were used in the experiments.

#### Cytofluorimetric Analysis

The immunophenotypic characterization of patient-derived fibroblasts was conducted by using cytofluorimetric analysis. Subconfluent cell cultures were washed with PBS, gently detached using trypsin–EDTA solution, and resuspended in complete medium. After centrifugation (1500 rpm for 5 min), the cells were washed in PBS to remove trypsin/EDTA, resuspended in 4% paraformaldehyde solution (in PBS), and incubated at room temperature for 20 min, with gentle shaking at 300 rpm. The fixed cells were centrifuged, resuspended in PBS containing 0.5% BSA, and washed. After centrifugation, the cells were resuspended in 100 μL of primary antibody in PBS with 0.5% BSA and incubated at 4 °C overnight. The primary antibodies used in this study were anti-CD90-PE conjugated antibody (Myltenyi Biotec, REA897, 1:80), anti-CD326-APC conjugated antibody (Myltenyi Biotec, REA764, 1:80) and rabbit anti-αVβ6 antibody (bs-5791R, Bioss Antibodies, 1:40). After incubation, the cells exposed to rabbit anti-αVβ6 antibody were washed and incubated for 1 h at 4 °C with a specific goat antirabbit FITC (no. 24549933, ImmunoTools, 1:100). Finally, each cell suspension was washed with PBS, centrifuged, and resuspended in 300 μL of PBS for FACS analysis.

#### Cell Internalization

The integrin-mediated cell internalization of conjugate **1** and free nintedanib was determined by exploiting the intrinsic fluorescence of the nintedanib moiety (λ_ex_ = 405 nm; λ_em_ = 480 nm).^29^ Intracellular fluorescence activity of 5 μM nintedanib was measured by flow cytometry. Fluorescence emission was analyzed using the BD Brilliant Violet 421 channel (450/50 nm bandpass filter) for the 405 nm laser.

Briefly, 150,000 cells were seeded in T25 flasks in complete culture medium and treated for 24 h with nintedanib or compound **1** at 5 μM concentration. After incubation, the cells were washed with PBS, gently detached using trypsin–EDTA solution, and resuspended in complete medium. After centrifugation (1500 rpm for 5 min), the cells were washed in PBS and resuspended in 300 μL of PBS for FACS analysis.

#### Western Blotting Analysis

IPF cells were plated in T25 flasks (500,000 cells/flask). The next day, the cells were treated for 24 h with nintedanib or different compounds (nintedanib, **1**, **3**, or nintedanib + **3**) at 5 μM concentration. After incubation, the cells were washed twice with cold PBS and lysed in 80 μL of RIPA lysis buffer (Merck Millipore, Vimodrone, Milan, Italy), containing 100 μM phenylmethylsulfonyl fluoride (PMSF) (Sigma-Aldrich) serine protease inhibitor, sodium orthovanadate (Na_3_VO_4_, Sigma-Aldrich) tyrosine phosphatase inhibitor, and a generic protease inhibitor diluted 1:1000 (Pierce Protease Inhibitor Mini, Thermo Fisher Scientific).

The protein content in each sample was quantified using the Bradford colorimetric assay, and 56 μg of total proteins was prepared for SDS-PAGE analysis using Bolt LDS Sample Buffer 4× (Novex, Life Technologies) with 10% Bolt Sample Reducing Agent 10× (Novex, Life Technologies). Protein samples were denatured at 90 °C for 5 min and separated on Bolt Bis-Tris Plus gels with 4–12% precast polyacrylamide gels (Life Technologies, Monza, Italy). Fractionated proteins were transferred from the gel to a PVDF nitrocellulose membrane by using the iBlot 2 system (Life Technologies, Monza, Italy). Membranes were blocked for 1 h at room temperature with Odyssey blocking buffer (Dasit Science, Cornaredo, MI, Italy). Next, the membranes were probed at 4 °C overnight with appropriate primary antibodies diluted in a solution of 1:1 Odyssey blocking buffer/T-PBS buffer, washed four times with PBS–Tween 0.1% solution, and probed with the secondary IRDye antibodies according to the manufacturer’s instructions. The primary antibodies were the following: rabbit anti-p44/42 MAPK (p-ERK1/2) (#9102, Cell Signaling 1:1000), mouse antivinculin (sc-25336, Santa Cruz 1:1000), and rabbit anti-TGF-β1 (Cat. No. orb11468, Biorbyt, Durham, North Carolina, 1:500). Next, the membranes were washed with PBS–Tween 0.1% solution, followed by incubation for 1 h at room temperature with the antirabbit IgG antibody Alexa Fluor 750 (Cod. No. A21039; Invitrogen, Monza, Italy) or the antimouse IgG antibody Alexa Fluor 680 (Cod. No. A21057; Invitrogen, Monza, Italy), diluted 1:10000 in a PBS solution containing Tween and 5% BSA. The membranes were washed again with PBS–Tween 0.1% solution and then visualized using an Odyssey infrared imaging system (LI-COR, Bioscience). Each band detected was quantified by densitometric analysis, using ImageJ software, and normalized against vinculin expression as the housekeeper protein.

### *In Vivo* Experiments

#### Experimental Animals and Animal Care

The experiments were carried out on male C57Bl/6 mice aged 7–8 weeks, purchased from Envigo (San Pietro al Natisone, Udine, Italy). Our animal facility housed animals in groups of five per cage under conventional conditions. After delivery, the animals were acclimatized to our local vivarium conditions for 7–10 days (room temperature: 20–24 °C; relative humidity: 40–70%; 12 h light–dark cycle; food and water *ad libitum*). All required measures were taken to mitigate the animals’ distress or discomfort, and a designated veterinarian or trained technicians assessed pain levels daily using a Visual Analogue Scale (VAS) ranging from 0 to 10. Humane outcomes included dyspnea, weight loss ≥20%, and VAS ≥6.

#### Ethics

All experiments were conducted in accordance with Chiesi Farmaceutici S.p.A.’s intramural animal welfare practices for animal experimentation, as well as the European Directive 2010/63/UE, Italian D.Lgs 26/2014, the revised “Guide for the Care and Use of Laboratory Animals” (National Research Council Committee, US, 2011), and the ARRIVE guidelines.^53^ The T31–32 study was performed at Chiesi Farmaceutici, which is an AAALAC (Association for Assessment and Accreditation for Laboratory Animal Care) certified facility; all experimental animal procedures used in the study were approved by the Italian Ministry of Health (protocol number: 742/2022-PR) and the internal AWB.

#### Experimental Protocols

Two independent studies were performed for 51 mice. **Study #1** (16 mice) was performed for investigating the role of integrin αvβ6 in a BLM-induced lung fibrosis mouse model through fluorescence and gene expression analyses complemented by μCT imaging (Figure 3). Further, **Study #2** (35 mice) aimed at assessing the antifibrotic effects of treatment with PepNIN conjugate **1** and integrin ligand **11** alone, in comparison with nintedanib as the reference compound (Figure 4).

#### Animal Procedures

Bleomycin hydrochloride (Baxter) dissolved in 50 μL of saline was administered via a triple oropharyngeal aspiration (OA) under 2.5% isoflurane anesthesia to induce pulmonary fibrosis, as previously described.^37,38^ The negative control group received a triple OA with saline (Saline).

All mice in **Study #1** were imaged by μCT on days 14 and 21. On day 21, 4 Saline- and 4 BLM-treated mice were administered with 10 nmol/mouse fluorescent probe **2** by intravenous injection; after 6 h, they were culled, lungs were excised, and the fluorescence signal was detected by Fluorescence Molecular Tomography (FMT) and Odyssey DLx (LI-COR). Finally, gene expression analysis was performed on fresh lung tissues (4 Saline and 4 BLM groups both at days 14 and 21).

In **Study #2**, BLM-treated mice (29 of 35 mice) on day 14 were divided into 5 different groups (reported in Table S2, Supporting Information) and were daily treated with nintedanib (94 or 23 μmol/kg), PepNIN **1** (23 μmol/kg), integrin ligand **11** (23 μmol/kg), or vehicle (1% Tween 80 in ultrapure water) by oral gavage from day 14 to day 21. All mice were longitudinally monitored by μCT imaging on days 14 and 21, and subsequently they were sacrificed for the histological assessment of lung fibrosis.

#### Micro-CT Imaging Acquisition Protocol

Mice thoraxes were imaged using a Quantum GX Micro-CT scanner (Revvity, Inc. Waltham, MA) after anesthetic induction and maintenance with 2% isoflurane. Images were taken in free-breathing mice using the following parameters: an X-ray tube current of 88 A, an X-ray tube voltage of 90 kV, and a total angle of 360° over a scan period of 4 min. The detector was 736 × 588 pixels with a size of 0.2 mm. The reconstruction method automatically trimmed the pixels to produce a projection size of 512 × 512 pixels. Binning was not employed. Each animal was placed supine on the scanner bed, with its chest adjusted to fit inside the field of view. To detect the breathing pattern, pictures were captured in “high-speed” mode, allowing the recording of the gating signal through a region of interest (ROI) positioned over the animals’ diaphragm. Projections were collected in “list mode” (labeled as “00000”, “00001”, ···, “14′687”) during a single continuous gantry rotation. A window displayed the breathing pattern and position of the projections that would be used to reconstruct the inspiratory (P01) and expiratory (P02) volumes at the end of each acquisition. The acquisition duration was 4 min, and around 900 projections (both P01 and P02) were automatically selected and used for the reconstruction of the two data sets, with the option to change the thresholds to select the most suited projections. However, our highly controlled anesthetic protocol^39,54^ enabled us to maintain constant breathing rates (100–120 bpm). Two stacks of 512 cross-sectional images were automatically reconstructed into two 3D data sets corresponding to the inspiratory and expiratory breathing phases (end-inspiration, P01; end-expiration, P02), with a 50 μm isotropic reconstructed voxel size, using a filtered back-projection algorithm with a Ram-Lak filter. The micro-CT scanner was calibrated monthly using standard phantoms for noise, homogeneity, low contrast, and resolution.^55^

#### Postprocessing of μCT Scans

A specially designed phantom with water and air was used to convert CT scans from gray levels to Hounsfield units (HU). The average gray levels of water and air were set to −1000 HU and 0 HU, respectively.^39^

A deep-learning-based segmentation model was used to handle the recovered data sets.^40^ Briefly, the model allowed the segmentation of the entire lung as well as of the left and right lungs independently for both the end-inspiration and end-expiration phases. The tool automatically calculated lung volumes (measured in mm^3^) and mean lung attenuation (MLA) densities (measured in Hounsfield units, HU) from each segmented volumes and also calculated aeration compartments (e.g., normo-, hypo-, and nonaerated areas) applying “HU preclinical ranges”.^39^ In this work, we specifically considered the %poorly aerated areas, which correspond to the combination of hypo- and nonaerated regions,^41^ and the %Gas measured at the end of the expiration (%Gas_Expiration_) as biomarkers discriminating regions with moderate-to-severe fibrosis and the content of air in the lung, respectively.^37^

### *Ex Vivo* Experiments

At the end point (on days 14 and 21, depending on the study), mice were killed after suffering an overdose of anesthesia and abdominal aortic hemorrhage. Lungs were then excised for *ex vivo* measurements.

#### Fluorescence Detection of Fluorescent Conjugate **2**

Lungs were imaged using an FMT 2500 *in vivo* imaging system (VisEn Medical, Inc., Bedford, MA).^42,43^ The collected fluorescence data were reconstructed with the FMT 2500 system software version 2.2 (PerkinElmer) for quantification of the fluorescence signal (in picomoles, pmol) within the lungs. Two representative lungs (one from Saline and one from BLM group at 21d) were also scanned with Odyssey DLx (LI-COR) at 800 nm wavelength with 21 μm resolution, to better visualize the localization of probe **2**.

#### Gene Expression by qPCR

RNA was extracted from 25 mg of fresh lung samples (right apical lobe) and retro-transcribed using the SuperScript IV VILO Master Mix (Invitrogen) according to the manufacturer’s instructions. A 2 μL aliquot of cDNA (1.25 ng/μL) was then mixed with TaqMan assay reagents (Mm01269869_m1 (Itgb6); Mm00801666_g1 (Col1a1); Mm00802300_m1 (Col3a1); Mm01256744_m1 (Fn1); Mm00437762_m1 (b2m)) and TaqMan Fast Advanced Master Mix. PCR was performed by using a QuantStudio 7 Flex real-time PCR system (Thermo Fisher Scientific). The cycle threshold (Ct) for each gene and sample was recorded, and gene expression was calculated based on 2^–ΔΔCt^, using β-2-microglobulin (B2m) as the reference housekeeping gene and Saline samples as the control group. Log_2_(fold change) obtained by comparison between BLM and Saline groups was reported in a heatmap chart.

#### Histological Assessment of Lung Fibrosis

The left lobe was stored for 24 h with 10% neutral buffered formalin and then included in paraffin. Using a rotary microtome (SLEE CUT 6062, SLEE medical, Mainz, Germany), a slice (5 μm) in the dorsal plane (coronal section) of each sample was cut. As directed by the manufacturer (Histo-Line Laboratories), the sections were stained with Picrosirius red to highlight collagen deposition and fibrotic alterations. The NanoZoomer S-60 digital slide scanner (Hamamatsu, Japan) was used to capture histological slides as whole slide images (WSI).

Each sample was evaluated in multiple 10× magnification areas, and trained histopathologists used the Ashcroft score (AS) scale^42,43^ to blindly rate the morphological changes. The distribution of Ashcroft score in each group is displayed as a violin plot.

### Statistical Analysis

#### *In Vitro* Studies on Human IPF Fibroblasts

Expression of cell-surface markers of mesenchymal or epithelial differentiation as well as the αvβ6 integrin receptor was assessed in IPF-derived cell populations isolated from at least three different patients, and one representative analysis is presented. Fluorescence intensity analysis in internalization studies was conducted in IPF-derived cell populations isolated from at least three different patients, and one representative result is shown. Western blotting analysis of TGF-β1 production and ERK1/2 phosphorylation was performed in three different patients. One representative result is shown. Densitometric data were analyzed using GraphPad Prism version 4 (GraphPad Software Inc., San Diego, CA). To evaluate statistically significant differences between group means across different treatment conditions, a two-way ANOVA (analysis of variance) was conducted. A Tukey’s honestly significant difference (HSD) posthoc test was then conducted.

#### *In Vivo* and *Ex Vivo* Studies

Statistical analyses were conducted using Prism 10 software (GraphPad Software Inc., San Diego, CA). Data are presented as mean ± SEM. A *t* test was used to compare fluorescence signals, and gene expression results were obtained for the Saline and BLM groups. For the other parameters, one- or two-way analysis of variance (ANOVA) was performed, followed by Dunnett’s multiple comparison posthoc test. Normality of data was assessed using the Shapiro–Wilk test, supplemented by visual inspection of QQ-plots. The Pearson correlation coefficient (*r* ^2^) was calculated when correlating fluorescence with μCT parameters (e.g., %poorly aerated tissue). A *p*-value of <0.05 (*) was considered statistically significant for all tests.

### Docking Studies

#### Protein Setup

The crystal structure of the extracellular domain of integrin αvβ6 in complex with the HGRGDLGRLKK undecapeptide of the TGF-β3 prodomain (PDB code: 4UM9)^44^ was used for docking studies. Docking was performed on the globular head of integrin because the headgroup of integrin has been identified in the X-ray structure as the ligand-binding region. The protein was truncated to residue sequences 1–439 for chain α (chain C of the crystal asymmetric unit) and 114–355 for chain β (chain D of the crystal asymmetric unit). According to the X-ray structure, the bivalent cation at MIDAS has been modeled as Mg^2+^ ions, whereas all other metal cations were modeled as Ca^2+^ ions. All water molecules were deleted except for the three water molecules coordinating the MIDAS cation and the single water molecule found around the ADMIDAS ion. The structure was then prepared by using the Protein Preparation Wizard of the graphical user interface Maestro and the OPLS2005 force field.^56^ Hydrogen bonds were optimized according to the exhaustive sampling option, and the entire complex was optimized by using a restrained minimization with convergence on heavy atoms to a RMSD (root-mean-square deviation) of 0.30 Å.

#### Ligand Docking

Automated docking calculations were performed by using Glide V90161 in the SP-peptide mode.^57^ The grids were generated for the RGD–integrin αvβ6 complex structure prepared as described in the Protein Setup section. The center of the grid-enclosing box was defined by the center of the bound ligand. (The GRGDLGRL octapeptide of the TGF-β3 prodomain was considered.) For the grid generation step, the size of the inner cubic box for placing the ligand center was set to 12 Å, and a value of 48 Å was used for the outer cubic box. For docking calculations, the GlideScore function was used to select 10 poses for each ligand after a postminimization step. The flexible docking option was selected, and the SP-peptide modality was used with the “penalize nonplanar amide torsions” option for amide bonds. Ring and nitrogen-inversion samplings were disabled. No Epik state penalty was added to the docking score. Ionized carboxylate and protonated guanidinium groups have been employed for the cyclic RGD peptide while maintaining neutral proline and piperazine basic moieties. The molecule size exceeds the maximum supported for generating protonation states by the Epik module.^58^

## Supplementary Material



## Notes

General information, materials, synthetic procedures, and characterization data of intermediate products; HPLC trace and HRMS spectra of **2**; *in vitro* plasma stability of **1** and **2**; solid-phase assay binding curves; *in vivo* experimental groups and body weight monitoring; docking studies of **1**; and statistical analysis (PDF)

## ◆

Department of Chemistry, Aarhus University, Aarhus DK-8000, Denmark

## Abbreviations

ADC: antibody–drug conjugate
Amp: *cis*-4-amino-l-proline
BLM: bleomycin
CT: computed tomography
DIPEA: diisopropylethylamine
DMF: dimethylformamide
ECM: extracellular matrix
EMT: epithelial-to-mesenchymal transition
ERK: extracellular signal-regulated kinase
FGF-2: fibroblast growth factor-2
HATU: hexafluorophosphate azabenzotriazole tetramethyl uranium
HOAt: 1-hydroxy-7-azabenzotriazole
IPF: idiopathic pulmonary fibrosis
LAP: latency-associated peptide
LC-MS: liquid chromatography–mass spectrometry
MAPK: mitogen-activated protein kinase
MIDAS: metal-ion-dependent adhesion site
NIR: near-infrared
PDC: peptide–drug conjugate
PepNIN: peptide–nintedanib conjugate
PET: positron emission tomography
PDGF: platelet-derived growth factor
RGD: arginine-glycine-aspartic acid
RP-HPLC: reverse-phase high-performance liquid chromatography
SMDC: small molecule–drug conjugate
SPPS: solid-phase peptide synthesis
TFA: trifluoroacetic acid
TIS: triisopropylsilane
TGF-β: transforming growth factor-β
TKI: tyrosine kinase inhibitor
VEGFR: vascular growth factor receptor

## References

[1] Lederer D. J., Martinez F. J. (2018). Idiopathic Pulmonary
Fibrosis. N. Engl. J. Med..

[2] Kolb M., Bonella F., Wollin L. (2017). Therapeutic
targets in idiopathic
pulmonary fibrosis. Resp. Med..

[3] Sgalla G., Iovene B., Calvello M., Ori M., Varone F., Richeldi L. (2018). Idiopathic pulmonary fibrosis: pathogenesis
and management. Respir. Res..

[4] Sofia C., Comes A., Sgalla G., Richeldi L. (2023). An update on emerging
drugs for the treatment of idiopathic pulmonary fibrosis: a look towards
2023 and beyond. Expert Opin. Emerging Drugs.

[5] Bonella F., Spagnolo P., Ryerson C. (2023). Current and
Future Treatment Landscape
for Idiopathic Pulmonary Fibrosis. Drugs.

[6] Sofia C., Comes A., Sgalla G., Richeldi L. (2024). Promising advances
in treatments for the management of idiopathic pulmonary fibrosis. Expert Opin. Pharmacother..

[7] Confalonieri P., Volpe M. C., Jacob J., Maiocchi S., Salton F., Ruaro B., Confalonieri M., Braga L. (2022). Regeneration or Repair?
The Role of Alveolar Epithelial Cells in the Pathogenesis of Idiopathic
Pulmonary Fibrosis (IPF). Cells.

[8] Rieder F., Nagy L. E., Maher T. M., Distler J. H. W., Kramann R., Hinz B., Prunotto M. (2025). Fibrosis:
cross-organ biology and
pathways to development of innovative drugs. Nat. Rev. Drug Discovery.

[9] Grimminger F., Gunther A., Vancheri C. (2015). The role of tyrosine
kinases in the
pathogenesis of idiopathic pulmonary fibrosis. Eur. Respir. J..

[10] Yu W.-K., Chen W.-C., Su V. Y.-F., Shen H.-C., Wu H.-H., Chen H., Yang K.-Y. (2022). Nintedanib Inhibits
Endothelial Mesenchymal
Transition in Bleomycin-Induced Pulmonary Fibrosis via Focal Adhesion
Kinase Activity Reduction. Int. J. Mol. Sci..

[11] Corte T., Bonella F., Crestani B., Demedts M. G., Richeldi L., Coeck C., Pelling K., Quaresma M., Lasky J. A. (2015). Safety,
tolerability and appropriate use of nintedanib in idiopathic pulmonary
fibrosis. Respir. Res..

[12] Tada A., Minami T., Kitai H., Higashiguchi Y., Tokuda M., Higashiyama T., Negi Y., Horio D., Nakajima Y., Otsuki T., Mikami K., Takahashi R., Nakamura A., Kitajima K., Ohmuraya M., Kuribayashi K., Kijima T. (2023). Combination therapy with anti-programmed cell death
1 antibody plus angiokinase inhibitor exerts synergistic antitumor
effect against malignant mesothelioma via tumor microenvironment modulation. Lung Cancer.

[13] Massagué J., Sheppard D. (2023). TGF-β signaling in health and
disease. Cell.

[14] Koivisto L., Bi J., Häkkinen L., Larjava H. (2018). Integrin αvβ6:
Structure, function and role in health and disease. Int. J. Biochem. Cell Biol..

[15] Hatley R. J. D., Macdonald S. J. F., Slack R. J., Le J., Ludbrook S. B., Lukey P. T. (2018). An αv-RGD
Integrin Inhibitor
Toolbox: Drug Discovery Insight, Challenges and Opportunities. Angew. Chem., Int. Ed..

[16] Slack R. J., Macdonald S. J. F., Roper J. A., Jenkins R. G., Hatley R. J. D. (2022). Emerging
therapeutic opportunities for integrin inhibitors. Nat. Rev..

[17] Decaris M. L., Schaub J. R., Chen C., Cha J., Lee G. G., Rexhepaj M., Ho S. S., Rao V., Marlow M. M., Kotak P., Budi E. H., Hooi L., Wu J., Fridlib M., Martin S. P., Huang S., Chen M., Muñoz M., Hom T. F., Wolters P. J., Desai T. J., Rock F., Leftheris K., Morgans D. J., Lepist E.-I., Andre P., Lefebvre E. A., Turner S. M. (2021). Dual inhibition
of αvβ6 and αvβ1 reduces fibrogenesis in lung
tissue explants from patients with IPF. Respir.
Res..

[18] Lancaster L. H., Cottin V., Ramaswamy M., Goldin J. G., Kim G. H. J., Bellini J., Jurek M., Decaris M., Cosgrove G. P., Lefebvre E., Flaherty K. R. (2023). PLN-74809
Shows Favorable Safety
and Tolerability and Indicates Antifibrotic Activity in a Phase 2a
Study for the Treatment of Idiopathic Pulmonary Fibrosis [abstract]. Am. J. Respir. Crit. Care Med..

[19] Raghu G., Mouded M., Chambers D. C., Martinez F. J., Richeldi L., Lancaster L. H., Hamblin M. J., Gibson K. F., Rosas I. O., Prasse A., Zhao G., Serenko M., Novikov N., McCurley A., Bansal P., Stebbins C., Arefayene M., Ibebunjo S., Violette S. M., Gallagher D., Behr J. (2022). A Phase IIb Randomized Clinical Study of an Anti-αvβ6
Monoclonal Antibody in Idiopathic Pulmonary Fibrosis. Am. J. Respir. Crit. Care Med..

[20] Roy A., Shi L., Chang A., Dong X., Fernandez A., Kraft J. C., Li J., Le V. Q., Winegar R. V., Cherf G. M., Slocum D., Poulson P. D., Casper G. E., Vallecillo-Zúniga M. L., Valdoz J. C., Miranda M. C., Bai H., Kipnis Y., Olshefsky A., Priya T., Carter L., Ravichandran R., Chow C. M., Johnson M. R., Cheng S., Smith M. K., Overed-Sayer C., Finch D. K., Lowe D., Bera A. K., Matute-Bello G., Birkland T. P., DiMaio F., Raghu G., Cochran J. R., Stewart L. J., Campbell M. G., Van Ry P. M., Springer T., Baker D. (2023). De novo design of highly
selective miniprotein inhibitors of integrins αvβ6 and
αvβ8. Nat. Commun..

[21] Battistini L., Bugatti K., Sartori A., Curti C., Zanardi F. (2021). RGD Peptide-Drug
Conjugates as Effective Dual Targeting Platforms: Recent Advances. Eur. J. Org. Chem..

[22] Lyon R. P., Jonas M., Frantz C., Trueblood E. S., Yumul R., Westendorf L., Hale C. J., Stilwell J. L., Yeddula N., Snead K. M., Kumar V., Patilea-Vrana G. I., Klussman K., Ryan M. C. (2023). SGN-B6A: A New Vedotin Antibody–Drug
Conjugate Directed to Integrin Beta-6 for Multiple Carcinoma Indications. Mol. Cancer Ther..

[23] Pang X., He X., Qiu Z., Zhang H., Xie R., Liu Z., Gu Y., Zhao N., Xiang Q., Cui Y. (2023). Targeting integrin
pathways: mechanisms and advances in therapy. Signal Transduction Targeted Ther..

[24] Steiger K., Quigley N. G., Groll T., Richter F., Zierke M. A., Beer A. J., Weichert W., Schwaiger M., Kossatz S., Notni J. (2021). There is a world beyond αvβ3-integrin:
Multimeric ligands for imaging of the integrin subtypes αvβ6,
αvβ8, αvβ3, and α5β1 by positron
emission tomography. EJNMMI Res..

[25] Kimura R. H., Iagaru A., Guo H. H. (2023). Mini review
of first-in-human integrin
αvβ6 PET tracers. Front. Nucl. Med..

[26] Hiroyama S., Matsunaga K., Ito M., Iimori H., Tajiri M., Nakano Y., Shimosegawa E., Abe K. (2022). Usefulness of ^18^F-FPP-RGD_2_ PET in pathophysiological
evaluation
of lung fibrosis using a bleomycin-induced rat model. Eur. J. Nucl. Med. Mol. Imaging.

[27] Dean T. T., Jelú-Reyes J., Allen A. C., Moore T. W. (2024). Peptide–Drug
Conjugates: An Emerging Direction for the Next Generation of Peptide
Therapeutics. J. Med. Chem..

[28] Bugatti K., Bruno A., Arosio D., Sartori A., Curti C., Augustijn L., Zanardi F., Battistini L. (2020). Shifting Towards
αvβ6 Integrin Ligands Using Novel Aminoproline-Based Cyclic
Peptidomimetics. Chem. - Eur. J..

[29] Bugatti K., Andreucci E., Monaco N., Battistini L., Peppicelli S., Ruzzolini J., Curti C., Zanardi F., Bianchini F., Sartori A. (2022). Nintedanib-Containing Dual Conjugates
Targeting αVβ6 Integrin and Tyrosine Kinase Receptors
as Potential Antifibrotic Agents. ACS Omega.

[30] Andreucci E., Bugatti K., Peppicelli S., Ruzzolini J., Lulli M., Calorini L., Battistini L., Zanardi F., Sartori A., Bianchini F. (2023). Nintedanib-αVβ6
Integrin Ligand Conjugates Reduce TGFβ-Induced EMT in Human
Non-Small Cell Lung Cancer. Int. J. Mol. Sci..

[31] Roth G. J., Heckel A., Colbatzky F., Handschuh S., Kley J., Lehmann-Lintz T., Lotz R., Tontsch-Grunt U., Walter R., Hilberg F. (2009). Design, Synthesis,
and Evaluation
of Indolinones as Triple Angiokinase Inhibitors and the Discovery
of a Highly Specific 6-Methoxycarbonyl-Substituted Indolinone (BIBF
1120). J. Med. Chem..

[32] Maji S., Barman S., Panda G. (2023). An Efficient
Approach Towards the
Synthesis of Nintedanib. ChemistrySelect.

[33] de
Valk K. S., Deken M. M., Handgraaf H. J. M., Bhairosingh S. S., Bijlstra O. D., van Esdonk M. J., Terwisscha van Scheltinga A. G. T., Valentijn A. R. P. M., March T. L., Vuijk J., Peeters K. C. M. J., Holman F. A., Hilling D. E., Mieog J. S. D., Frangioni J. V., Burggraaf J., Vahrmeijer A. L. (2020). First-in-Human Assessment of cRGD-ZW800–1,
a Zwitterionic, Integrin-Targeted, Near-Infrared Fluorescent Peptide
in Colon Carcinoma. Clin. Cancer Res..

[34] Choi H. S., Nasr K., Alyabyev S., Feith D., Lee J. H., Kim S. H., Ashitate Y., Hyun H., Patonay G., Strekowski L., Henary M., Frangioni J. V. (2011). Synthesis
and in vivo fate of zwitterionic near-infrared fluorophores. Angew. Chem., Int. Ed..

[35] Hyun H., Bordo M. W., Nasr K., Feith D., Lee J. H., Kim S. H., Ashitate Y., Moffitt L. A., Rosenberg M., Henary M., Choi H. S., Frangioni J. V. (2012). cGMP-Compatible
preparative scale synthesis of near-infrared fluorophores. Contrast Media Mol. Imaging.

[36] Kapp T. G., Rechenmacher F., Neubauer S., Maltsev O. V., Cavalcanti-Adam E. A., Zarka R., Reuning U., Notni J., Wester H.-J., Mas-Moruno C., Spatz J., Geiger B., Kessler H. (2017). A Comprehensive
Evaluation of the Activity and Selectivity Profile of Ligands for
RGD-binding Integrins. Sci. Rep..

[37] Buccardi M., Ferrini E., Pennati F., Vincenzi E., Ledda R. E., Grandi A., Buseghin D., Villetti G., Sverzellati N., Aliverti A., Stellari F. F. (2023). A fully
automated micro CT deep learning
approach for precision preclinical investigation of lung fibrosis
progression and response to therapy. Respir.
Res..

[38] Khalajzeyqami Z., Grandi A., Ferrini E., Ravanetti F., Leo L., Mambrini M., Giardino L., Villetti G., Stellari F. F. (2022). Pivotal
role of micro-CT technology in setting up an optimized lung fibrosis
mouse model for drug screening. PLoS One.

[39] Ferrini, E. ; Buccardi, M. ; Stellari, F. F. In Vivo Micro-CT Imaging for Quantitative Longitudinal Assessment of Pulmonary Diseases in Small Animals. In Target Identification and Validation in Drug Discovery; Moll, J. ; Carotta, S. , Eds.; Methods in Molecular Biology; Springer: New York, 2025; Vol. 2905, pp 207–232. 10.1007/978-1-0716-4418-8_14 40163308

[40] Vincenzi E., Fantazzini A., Basso C., Barla A., Odone F., Leo L., Mecozzi L., Mambrini M., Ferrini E., Sverzellati N., Stellari F. F. (2022). A fully automated deep learning pipeline for micro-CT-imaging-based
densitometry of lung fibrosis murine models. Respir. Res..

[41] Mecozzi L., Mambrini M., Ruscitti F., Ferrini E., Ciccimarra R., Ravanetti F., Sverzellati N., Silva M., Ruffini L., Belenkov S., Civelli M., Villetti G., Stellari F. F. (2020). In-vivo
lung fibrosis staging in a bleomycin-mouse model: a new micro-CT guided
densitometric approach. Sci. Rep..

[42] Stellari F., Sala A., Ruscitti F., Carnini C., Mirandola P., Vitale M., Civelli M., Villetti G. (2015). Monitoring inflammation
and airway remodeling by fluorescence molecular tomography in a chronic
asthma model. J. Transl. Med..

[43] Stellari F. F., Ruscitti F., Pompilio D., Ravanetti F., Tebaldi G., Macchi F., Verna A. E., Villetti G., Donofrio G. (2017). Heterologous matrix metalloproteinase
gene promoter
activity allows in vivo real-time imaging of bleomycin-induced lung
fibrosis in transiently transgenized mice. Front.
Immunol..

[44] Dong X., Hudson N. E., Lu C., Springer T. A. (2014). Structural determinants
of integrin β-subunit specificity for latent TGF-β. Nat. Struct. Mol. Biol..

[45] Xiong J. P., Stehle T., Zhang R., Joachimiak A., Frech M., Goodman S. L., Arnaout M. A. (2002). Crystal Structure
of the Extracellular Segment of Integrin αVβ3 in Complex
with an Arg-Gly-Asp Ligand. Science.

[46] Springer T. A., Zhu J., Xiao T. (2008). Structural
basis for distinctive recognition of fibrinogen
γC peptide by the platelet integrin αIIbβ3. J. Cell Biol..

[47] Xia W., Springer T. A. (2014). Metal ion and ligand
binding of integrin α5β1. Proc.
Natl. Acad. Sci. U.S.A..

[48] Wang J., Su Y., Iacob R. E., Engen J. R., Springer T. A. (2019). General structural
features that regulate integrin affinity revealed by atypical αVβ8. Nat. Commun..

[49] Civera M., Arosio D., Bonato F., Manzoni L., Pignataro L., Zanella S., Gennari C., Piarulli U., Belvisi L. (2017). Investigating
the Interaction of Cyclic RGD Peptidomimetics with αvβ6
Integrin by Biochemical and Molecular Docking Studies. Cancers.

[50] Sime P., Jenkins G. (2022). Goldilocks and the three trials: clinical trials targeting
the αvβ6 integrin in idiopathic pulmonary fibrosis. Am. J. Respir. Crit. Care Med..

[51] Keenan, C. Pliant Therapeutics Provides Update on BEACON-IPF, a Phase 2b/3 Trial in Patients with Idiopathic Pulmonary Fibrosis; Pliant Therapeutics, Inc., 2025. www.PliantRx.com.

[52] The TKI activity of compound **1** against human recombinant VEGFR was previously reported; IC_50_ = 10 nM for **1**; IC_50_ = 7 nM for nintedanib; see ref 29.

[53] du
Sert N. P., Hurst V., Ahluwalia A., Alam S., Avey M. T., Baker M., Browne W. J., Clark A., Cuthill I. C., Dirnagl U., Emerson M., Garner P., Holgate S. T., Howells D. W., Karp N. A., Lazic S. E., Lidster K., MacCallum C. J., Macleod M., Pearl E. J., Petersen O. H., Rawle F., Reynolds P., Rooney K., Sena E. S., Silberberg S. D., Steckler T., Würbel H. (2020). The ARRIVE guidelines 2.0: Updated
guidelines for reporting animal research. PLoS
Biol..

[54] Ferrini E., Mecozzi L., Corsi L., Ragionieri L., Donofrio G., Stellari F. F. (2020). Alfaxalone and Dexmedetomidine
as
an Alternative to Gas Anesthesia for Micro-CT Lung Imaging in a Bleomycin-Induced
Pulmonary Fibrosis Murine Model. Front. Vet.
Sci..

[55] Mambrini M., Mecozzi L., Ferrini E., Leo L., Bernardi D., Grandi A., Sverzellati N., Ruffini L., Silva M., Stellari F. F. (2022). The importance of
routine quality control for reproducible
pulmonary measurements by in vivo micro-CT. Sci. Rep..

[56] Maestro; Schrödinger, LLC: New York, NY, USA, 2021.

[57] Glide, version 90161; Schrödinger, LLC: New York, NY, USA, 2021.

[58] Epik, version 5.5161; Schrödinger, LLC: New York, NY, USA, 2021.

