# Supplementary material for: Antifibrotic Efficacy of a Nintedanib–Peptide Conjugate and Diagnostic Potential of a Fluorescent Companion Probe Targeting αVβ6 Integrin in Idiopathic Pulmonary Fibrosis
Source: ACS Pharmacol Transl Sci. 2025 Sep 17;8(10):3613–30. doi: 10.1021/acsptsci.5c00457 (PMC12519263; doi:10.1021/acsptsci.5c00457)
Supplement: Supplementary file 1 [file pt5c00457_si_001.pdf]

SUPPORTING INFORMATION

Antifibrotic Efficacy of a Nintedanib-Peptide Conjugate and Diagnostic Potential of a Fluorescent Companion Probe Targeting $\alpha_V\beta_6$ Integrin in Idiopathic Pulmonary Fibrosis

Kelly Bugatti,[a] Erica Ferrini,[b,c] Margherita Restori,[a] Costanza Bonfini,[d] Melissa Marchese,[e] Francesca Bianchini,[e] Sara Tomassetti,[f] Andrea Maurizio,[g] Monica Baiula,[g] Lucia Battistini,[a] Enrico Marcantonio,[a,†] Claudio Curti,[a] Monica Civera,[h] Laura Belvisi,[h] Andrea Sartori[a,] Franco F. Stellari[b,]*, Franca Zanardi[a,]**

[a]Department of Food and Drug, University of Parma, 43124, Parma, Italy; [b]Molecular Imaging Facility, Experimental Pharmacology & Translational Science Department, Chiesi Farmaceutici S.p.A., 43122, Parma, Italy; [c]ANTHEM (AdvaNced Technologies for Human-centrEd Medicine), Spoke 3, Milan, Italy; [d]Department of Veterinary Science, University of Parma, 43126, Parma, Italy; [e]Department of Experimental and Clinical Biomedical Sciences "Mario Serio", University of Florence, 50134, Florence, Italy; [f]Department of Clinical and Experimental Medicine, University of Florence, 50134, Florence, Italy; [g]Department of Pharmacy and Biotechnology, University of Bologna, 40126, Bologna, Italy; [h]Department of Chemistry, University of Milan, 20133, Milan, Italy

*Corresponding authors: Andrea Sartori, e-mail: andrea.sartori@unipr.it; Franco F. Stellari, e-mail: fb.stellari@chiesi.com; Franca Zanardi, e-mail: franca.zanardi@unipr.it

Table of Contents

1. Chemistry: General information, materials, synthetic procedures and characterization data, HPLC trace and HRMS spectrum of compound **2** (**Figures S1-S2**) S2

2. *In vitro* plasma stability of compounds **1** and **2** (**Table S1**) S7

3. *In vitro* biological studies: solid-phase binding curves (**Figure S3**) S9

4. *In vivo* experiments: experimental groups (**Table S2**), body weight monitoring in pharmacological Study #2 (**Figure S4**) S10

5. Docking studies of conjugate PepNIN **1** (**Figure S5**) S12

6. Statistical analysis S13

7. References S14

1. Chemistry

General. All chemicals were of the highest commercially available quality and were used without further purification. Solvents were dried by standard procedures and reactions requiring anhydrous conditions were performed under nitrogen or argon atmosphere. H-Gly-2-ClTrt resin was purchased from Novabiochem, (2*S*,4*S*)-Fmoc-4-amino-1-Boc-pyrrolidine-2-carboxylic acid from PolyPeptide and all other reagents from Fluorochem, Alfa Aesar, TCI, or Merck-Sigma-Aldrich. The automated flash chromatography and HPLC solvents respond to ACS standard and were used without further purification. Analytical thin layer chromatography (TLC) was performed on silica gel 60 F_{254} pre-coated plates with visualization under short-wavelength UV light and by dipping the plates with molybdate reagent (aqueous H_2SO_4 solution of ceric sulphate/ammonium molybdate) followed by heating. Flash column chromatography was performed using 40-63 μm silica gel and the indicated solvent mixtures. Automated flash column chromatography was carried out with the Biotage Isolera One system using Biotage KP-C18-HS cartridges (reverse phase). ESI-mass spectra were recorded on UHPLC/ESI-MS system (ACQUITY Ultra Performance LC; ESI, positive ions, Single Quadrupole analyzer) and are reported in the form of (*m/z*). HPLC purifications were performed on a Shimadzu Nexera X2 apparatus (UV detection) equipped with C18-10 μm columns (Discovery BIO Wide Pore 10 × 250 mm or 21.2 × 250 mm). Routine NMR spectra were recorded on Avance 400 (Bruker) NMR spectrometers. Chemical shifts (δ) are reported in parts per million (ppm) with TMS ($CDCl_3$), CD_2HOD, and HOD resonance peaks set at 0.00, 3.31, and 4.80 ppm, respectively. Multiplicities are indicated as s (singlet), d (doublet), t (triplet), q (quartet), m (multiplet), and b (broad). Coupling constants, *J*, are reported in Hertz. ^{1}H assignments are corroborated by 1D and 2D experiments (^{1}H-^{1}H COSY, ^{1}H-^{1}H TOCSY). High resolution mass analysis (ESI) was performed on LTQ ORBITRAP XL Thermo apparatus. Purity of the final compounds was checked by Shimadzu Nexera X2 (UV detection) equipped with an analytical column (C18-10 μm column Discovery BIO Wide Pore 4.6 × 250 mm).

Materials. Protected azido-terminating cyclopeptide **prot-3**, the corresponding deprotected cyclopeptide **3**, the alkyne-terminating compound **4**, as well as its precursors **5** and **6**, were prepared and fully characterized according to previously reported procedures.[1] Chlorocyclohexene **7** was prepared by double Vilsmeier-Haak formylation of commercial cyclohexanone and subsequent double condensation with commercial aniline by following a reported procedure.[2]

Protected cyclopeptide **prot-11** and the corresponding deprotected ligand **11**, used as cyclopeptide reference compound in *in vivo* studies, were prepared by following a mixed solid-phase/in-solution peptide synthesis according to a previously reported procedure.[3]

Synthetic Procedures and Characterization Data.

*Synthesis of Indolium Sulfonate **8**.* The synthesis of known compound **8** was carried out by adopting slightly modified conditions taken from a reported two-step procedure.[2] Briefly, 4-hydrazinebenzensolfonic acid (600 mg, 3.18 mmol, 1 eq) was suspended in glacial acetic acid (3 mL) in a Schlenk tube. Then, 3-methyl-2-butanone (472 µL, 4.45 mmol, 1.4 eq) was added to the suspension. The reaction was left stirring at 110 °C in an inert atmosphere for 20 h. Then, the reaction mixture was diluted with EtOAc and filtered. The recovered pink solid was dissolved in 10 mL of MeOH/*i*PrOH (1:1), and NaOH (100 mg) was added. The reaction was left stirring at 100 °C for 30 min, then the solvent was removed under reduced pressure affording a brown solid (673.6 mg, 82%). The ^{1}H NMR and mass spectra coincided with the reported characterization data.[2] The intermediate compound (541.0 mg, 2.073 mmol, 1 eq), (3-bromopropyl)trimethylammonium bromide (1082.0 mg, 4.146 mmol, 2 eq) and NaI (62.1 mg, 0.416 mmol, 0.2 eq) were dissolved in acetonitrile (6 mL), and the resulting mixture was left stirring at 120 °C for 48 h. Then, the reaction was allowed to return at room temperature and isopropanol was added. The mixture was filtrated and washed with isopropanol (50 mL × 2) and diethyl ether (3 × 50 mL). The solid was recovered in methanol and methyl *tert*-butyl ether was added leading to the precipitation of product **8** as a pink solid (690.2 mg, 64% yield). ^{1}H NMR (400 MHz, D_2O) δ 8.02 (m, 1H), 7.94-7.88 (m, 1H), 7.86 (dd, J = 8.5, 1.8 Hz, 1H), 4.58 (t, J = 7.9, 2H), 3.74-3.60 (m, 2H), 3.28 (s, 3H), 3.17 (s, 9H), 2.54-2.40 (m, 2H), 1.55 (s, 6H). MS (ES$^+$): *m/z* 339.2 [M]$^+$.

*Synthesis of Compound **9**.* The synthesis of compound **9** was carried out by adopting a reported procedure.[1] In a round-bottomed flask equipped with a magnetic stir bar and kept under nitrogen atmosphere, compound **7** (32 mg, 0.089 mmol, 1 eq), compound **8** (96 mg, 0.18 mmol, 1.8 eq), and NaOAc (22 mg, 0.27 mmol, 3 eq) were suspended in dry EtOH (1.7 mL). The mixture was maintained at reflux conditions for 6 h under a nitrogen atmosphere. The solvent was then removed, and the solid was washed with CH_2Cl_2. The crude was suspended in methanol and filtrated, yielding to compound **9** as a bronze-green solid (70 mg, 70%). ^{1}H NMR (400 MHz, DMSO-d_6) δ 8.30 (d, J = 13.9 Hz, 2H), 7.84 (d, J = 1.6 Hz, 2H), 7.69 (dd, J = 8.3, 1.6 Hz, 2H),

7.44 (d, J = 8.3 Hz, 2H), 6.34 (d, J = 14.0 Hz, 2H), 4.26 (t, J = 6.5 Hz, 4H), 3.54-3.40 (m, 4H), 3.07 (s, 18H), 2.81-2.69 (m, 4H), 2.25-2.11 (m, 4H), 1.83 (m, 2H), 1.71 (s, 12H). MS (ES^+): *m/z* 813.3 $[M]^+$.

*Synthesis of Compound **10** (ZW800-1).* The synthesis of known compound **10** was carried out by adopting slight modifications of a reported procedure.[2] Commercial 3-(4-hydroxyphenyl)propanoic acid (204 mg, 1.23 mmol, 1 eq) and NaOH (98 mg, 2.5 mmol, 2 eq) were dissolved in H_2O (500 µL) and the resulting reaction mixture was left stirring for 2 h. The solvent was then removed, and the resulting white solid product (173 mg, 98% yield, the disodium salt of the starting material) was used for the subsequent step without further purification. This compound (9.5 mg, 0.045 mmol, 4 eq) and compound **9** (13.5 mg, 0.011 mmol, 1 eq) were dissolved in dry DMSO (400 µL) in a microwave tube. The reaction was carried out at 65 °C for 45 minutes, at 300 mW. Then diethyl ether (4 mL) was added to favor the precipitation of a solid, which was filtered and then purified by automated flash chromatography; solvent system: H_2O + 0.1% TFA (solvent A) and MeOH (solvent B); λnm detected: 220 nm/700 nm, method: from 100% solvent A to 100% solvent B furnishing product **10** as a bright-green solid (13.4 mg, 88% yield). ^1H NMR (400 MHz, CD_3OD) δ 8.03 (d, J = 14.0 Hz, 2H), 7.80-7.74 (m, 4H), 7.33 (d, J = 8.3 Hz, 2H), 7.26 (d, J = 8.6 Hz, 2H), 7.01 (d, J = 8.6 Hz, 2H), 6.24 (d, J = 14.1 Hz, 2H), 4.20 (t, J = 7.4 Hz, 4H), 3.62-3.51 (m, 4H), 3.16 (s, 18H), 2.84 (t, J = 7.2 Hz, 2H), 2.80-2.72 (m, 4H), 2.54 (t, J = 7.0, 2H), 2.33-2.18 (m, 4H), 2.09-1.96 (m, 2H), 1.34 (s, 12H). MS (ES+): *m/z* 472.3 $[M+H]^{2+}$.

*Synthesis of Compound **prot-12**.* In a round-bottomed flask equipped with a magnetic stir bar and kept under nitrogen atmosphere, protected cyclopeptide **prot-11** (30.4 mg, 0.024 mmol, 1 eq) was dissolved in a 1:1 MeOH/H_2O solution (1 mL), and zinc powder (15 mg, 0.225 mmol, 9 eq) and NH_4Cl (4 mg, 0.075 mmol, 0.4 eq) were added. The reaction was left stirring at 80 °C for 2 h, then the reaction was filtered, and the solvent was removed under reduced pressure. The crude was purified by automated flash chromatography (gradient: from 80:20 H_2O + 0.1% TFA/CH_3CN to 100% CH_3CN) giving product **prot-12** (25.6 mg, 79% yield) as a white solid. ^1H NMR (400 MHz, CD_3OD) δ 4.51 (m, 1H), 4.56 – 4.37 (m, 3H), 4.28 (t, J = 6.9 Hz, 1H), 4.13 (dd, J = 9.4, 6.2 Hz, 1H), 4.08 (d, J = 17.2 Hz, 1H), 3.91 (dd, J = 10.6, 6.4 Hz, 1H), 3.72 (d, J = 17.2 Hz, 1H), 3.41 (dd, J = 10.5, 5.5 Hz, 1H), 3.17 (m, 1H), 2.99 – 2.87 (m, 3H), 2.81 (dd, J = 16.1, 9.0 Hz, 1H), 2.69 (t, J = 6.6 Hz, 2H), 2.59 (s, 3H), 2.58 (s, 3H), 2.48 – 2.41 (m, 1H), 2.38 – 2.23 (m, 1H), 2.12 (s, 3H), 1.97 (m, 1H), 1.86 (t, J = 6.6 Hz, 2H), 1.91 – 1.52 (m, 13H), 1.48 (s, 9H), 1.42 (m, 2H), 1.33 (s,

6H), 1.31 (m, 3H), 1.01 (d, J = 6.6 Hz, 3H), 0.97– 0.92 (m, 6H), 0.90 (d, J = 6.2 Hz, 3H). MS (ES+): *m/z* 551.9 $[M+2H]^{2+}$.

*Synthesis of Conjugate **2**.* In a round-bottomed flask equipped with a magnetic stir bar and kept under nitrogen atmosphere, compound **10** (9 mg, 6.6 µmol, 1.5 eq) was dissolved in DMF (300 µL), and then HATU (3.37 mg, 0.0089 mmol, 2 eq), and DIPEA (9 µL, 0.0517 mmol, 11 eq) were sequentially added. The reaction was left stirring for 5 min and then **prot-12** (5.9 mg, 4.4 µmol, 1 eq) was added. The reaction was left stirring for 2 h, and then the reaction mixture was evaporated. The resulting crude mixture was purified by automated flash chromatography using the following solvent system: H_2O + 0.1% TFA (Solvent A) and ACN (Solvent B); λnm detected: 220 nm/700 nm; method: from 20% to 100% solvent B. A bright-green solid was recovered, which was treated with a solution of TFA/TIS/H_2O (95:2.5:2.5). The reaction was left stirring for 1.5 h; then the solvent was removed and the crude was purified with automated flash chromatography using the following solvent system: H_2O + 0.1% TFA (Solvent A) and ACN (Solvent B); λnm detected: 220 nm/700 nm; method: from 20% to 100% solvent B. Product **2** was obtained as a bright-green solid (4.7 mg, 55% two-step yield from **10**). ^{1}H NMR (400 MHz, CD_3OD) δ 8.08 (d, J = 13.8 Hz, 2H), 7.94 – 7.76 (m, 4H), 7.37 (dd, J = 8.7, 3.0 Hz, 2H), 7.28 (d, J = 8.4 Hz, 2H), 7.05 (dd, J = 8.5, 3.6 Hz, 2H), 6.28 (d, J = 14.9 Hz, 2H), 4.53 – 4.29 (m, 4H), 4.22 (m, 5H), 4.14 – 4.06 (m, 1H), 4.07 (d, J = 17.5 Hz, 1H), 3.94 – 3.84 (m, 1H), 3.81 (d, J = 17.5 Hz, 1H), 3.65 – 3.53 (m, 4H), 3.43 – 3.32 (m, 1H), 3.29 – 3.23 (m, 2H), 3.20 (s, 18H), 3.18 – 3.12 (m, 2H), 3.12 – 3.05 (m, 2H), 3.06 – 2.97 (m, 1H), 2.95 – 2.77 (m, 8H), 2.59 – 2.49 (m, 1H), 2.48 – 2.37 (m, 2H), 2.38 – 2.22 (m, 4H), 2.19 – 2.01 (m, 2H), 1.99 – 1.84 (m, 4H), 1.81 – 1.58 (m, 10H), 1.58 – 1.48 (m, 2H), 1.39 (s, 12H), 1.02 - 0.83 (m, 12H). HRMS (ES+) $C_{86}H_{126}N_{15}O_{17}S_2^+$ calcd for $[M+3H]^{3+}$ 569.3042; found 569.3024 $[M+3H]^{3+}$. Purity >98%. HPLC trace and HRMS spectrum in Figures S1 and S2.

HPLC trace for purity assessment of compound **2**

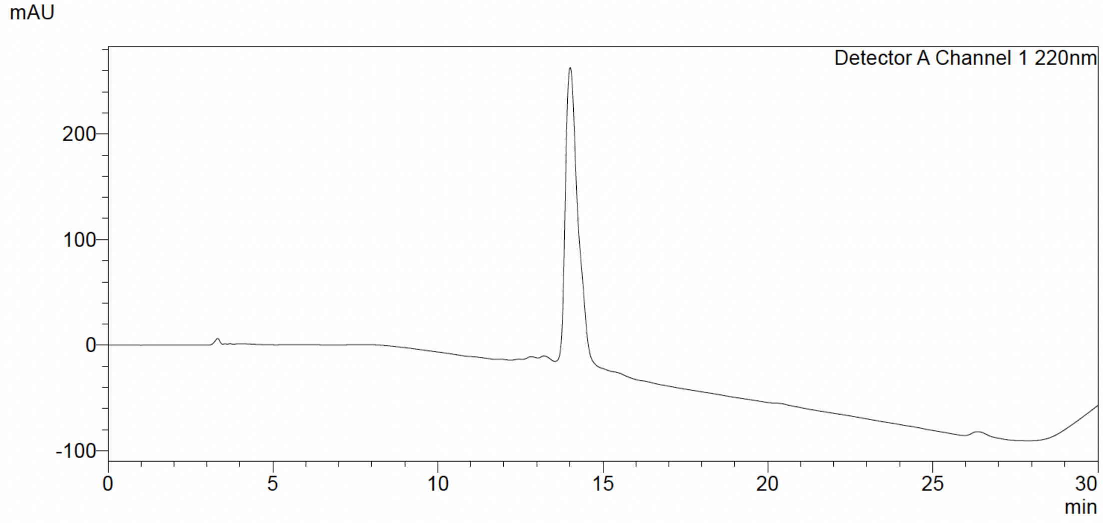

Figure S1. Purity assessment of compound **2** performed on an analytical C18-10 µm column (Discovery BIO Wide Pore 4.6 × 250 mm). Flow rate = 1.0 mL/min, λ detection = 700 nm. Mobile phase: solvent A (water + 0.1% trifluoroacetic acid) and solvent B (ACN). LC gradient: t = 0 min (B:10%); t=20 min (B:60%); t=22 min (B:60%); t=30 min (B:10%). t_R = 14.0 min.

HRMS of compound **2**

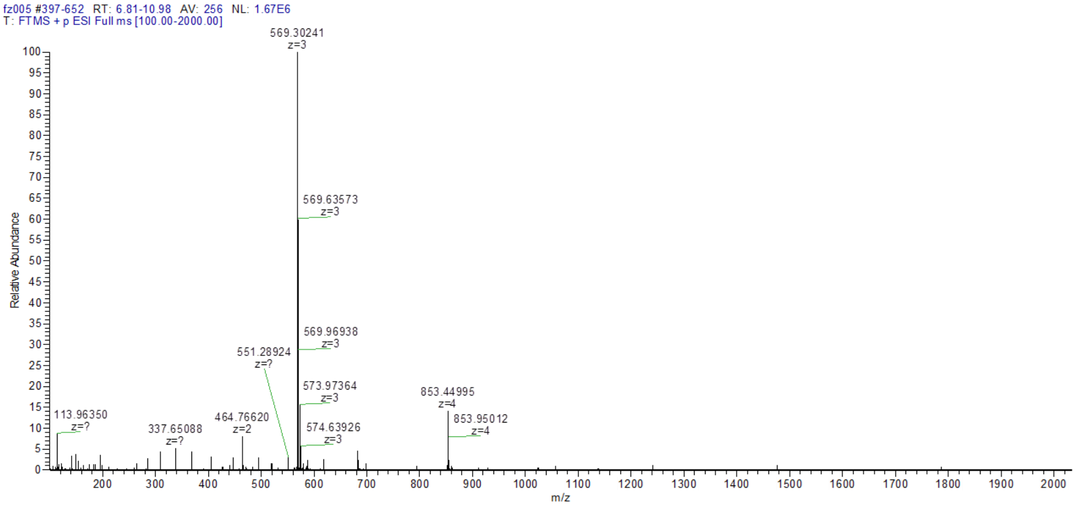

Figure S2. High resolution mass spectrum (ESI+) of compound **2** (LTQ ORBITRAP XL Thermo apparatus).

2. ***In vitro* plasma stability of compounds 1 and 2**

Stability of compounds **1** and **2** in mouse and human plasma was evaluated by incubating 5 µL of a 100 µM solution of each compound in DMSO with 400 µL of plasma and 95 µL of 100 mM phosphate buffered saline (PBS) pH 7.4 (final compound concentration: 1 µM; final DMSO concentration: 1%). Plasma samples were pre-incubated at 37 °C for 10 min before adding the compound. At different time points, an aliquot was withdrawn from each sample and deproteinized by addition of 100 µL of ice-cold acetonitrile containing the structurally related compound **13** as internal standard (Table S1). After a centrifugation step (14,000 g, 10 min, 4 °C), the supernatants were analyzed by LC-MS/MS. A multiple reaction monitoring (MRM) acquisition method in positive ion mode (ESI+) was set up to monitor the remaining percentages of test compound at different time points, if compared to time t = 0.

LC-MS/MS Analytical Method. A Thermo TSQ Quantum triple-quadrupole mass spectrometer (Thermo Italia, Milan, Italy) equipped with a heated electrospray (H-ESI) ion source and coupled to a Thermo Accela UHPLC system was employed for LC-MS/MS data acquisition and analysis. The LC separation occurred on a Waters XSelect HSS T3 column (3.5 µm, 2.1 × 100 mm; Waters, Milford, USA) at room temperature. The flow rate was 0.2 mL/min with a mobile phase consisting of solvent A (water + 0.1% formic acid) and solvent B (ACN + 0.1% formic acid). LC gradient was as follows: t= 0 min (A:95%; B:5%); t=8 min (A:20%; B:80%); t=10 min (A:20%; B:80%); t=10.5 min (A:95%; B:5%); t=12 min (A:95%; B:5%). H-ESI parameters were set as follows: probe middle (D) position; capillary temperature: 270 °C; spray voltage: 4.0 kV; sheath gas (N_2): 35 psi; auxiliary gas (N_2): 15 psi. Collision gas (Ar) at a pressure of 1.5 mtorr (1 torr = 133.3 Pa). For LC-MS/MS analysis, the following parent → product ion transitions were selected: compound **1**: *m/z* 487.3 $[M+3H]^{3+}$→ *m/z* 361.0 + *m/z* 658.2 + *m/z* 667.3 (Tube Lens (TL) 93 V; Collision Energy (CE): 18, 20, 19 eV); compound **2**: *m/z* 569.3 $[M+3H]^{3+}$→ *m/z* 113.8 + *m/z* 237.6 + *m/z* 324.8 (Tube Lens (TL) 107 V; Collision Energy (CE): 33, 26, 24 eV); compound **13** (Internal Standard): *m/z* 505.8 $[M+4H]^{4+}$→ *m/z* 251.8 + *m/z* 360.8 + *m/z* 650.2 (TL: 115 V; CE: 44, 20, 18 eV).

Table S1. Evaluation of *in vitro* stability of conjugated compounds **1** and **2** in mouse and human plasma

Compound	% Compound at 6h mouse plasma[a]	% Compound at 6h human plasma[a]
**1**	95 ± 4	96 ± 5
**2**	59 ± 3	51 ± 3

[a]Compound was incubated in 80% *v/v* mouse or human plasma, at 37°C. Mean values ± SD (n=3). are reported. The structurally related compound **13** reported below was used as an internal standard.

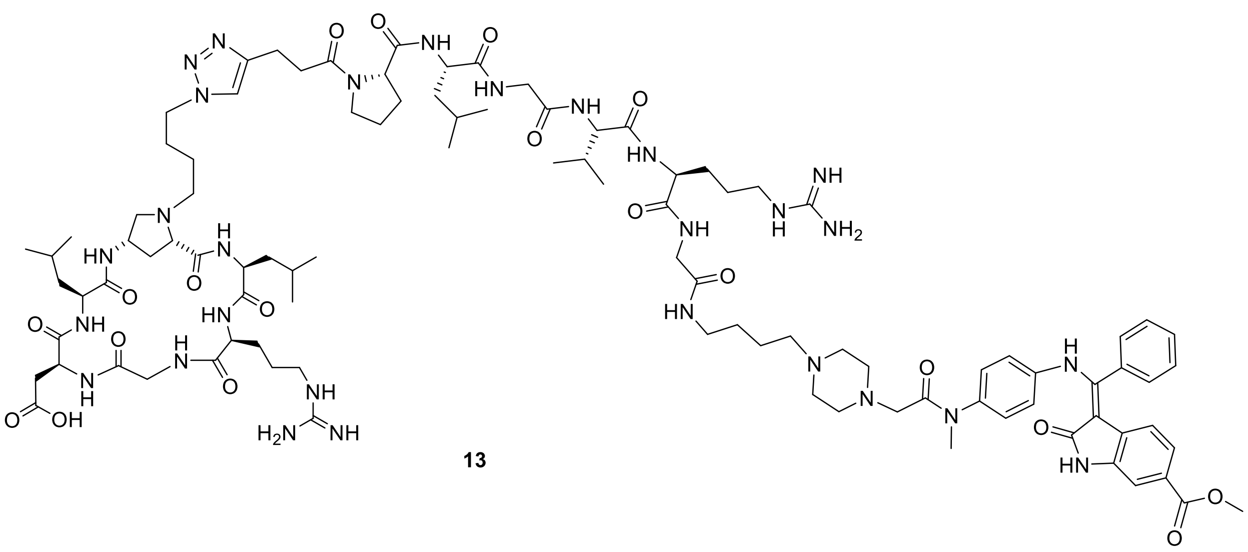

13

3. *In vitro* biological studies

Solid-phase binding curves

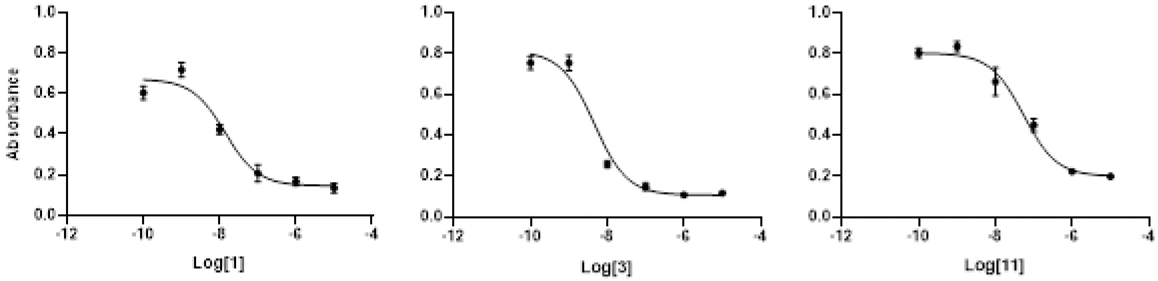

Figure S3. Binding curves deriving from solid-phase binding assays. Binding assay curves for αvβ6/LAP in the presence of increasing concentrations of compounds **1**, **3**, and **11**. Values represent the mean ± SD of three independent experiments carried out in triplicate.

4. *In vivo* experiments

Table S2. Experimental groups in Study #2

**Group/Treatment**	**N/Group**
Saline	6
BLM + Vehicle	8
BLM + NINT 94 µmol/kg	6
BLM + NINT 23 µmol/kg	5
BLM + PepNIN **1** 23 µmol/kg	5
BLM + **11** 23 µmol/kg	5

Body weight monitoring in pharmacological Study #2

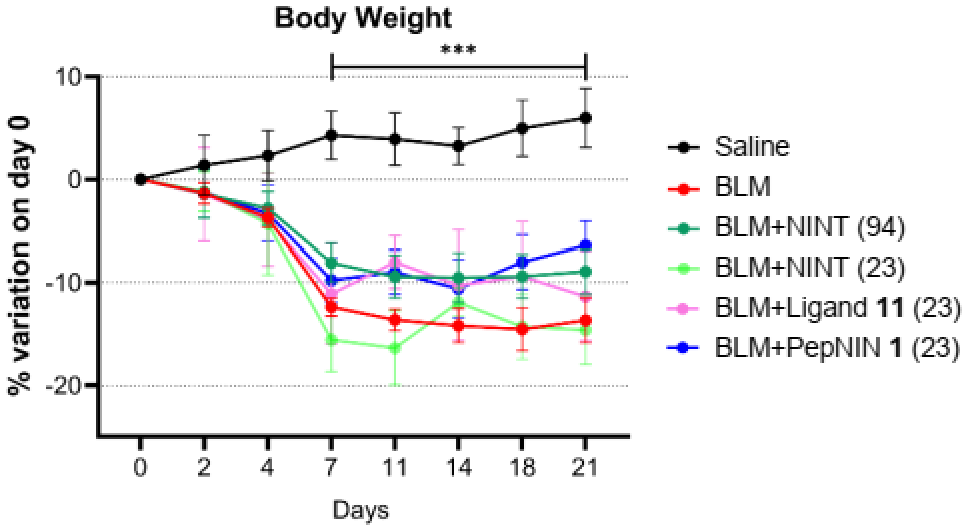

Figure S4. Body weight monitoring in pharmacological Study #2. The percentage of variation in body weight was reported for each treatment group compared to their value on day 0. Two-way ANOVA with Dunnett's test for multiple comparisons was utilized to compare body weight variation of each group *vs* BLM group at each timepoint, *** $p<0.001$.

5. Docking studies

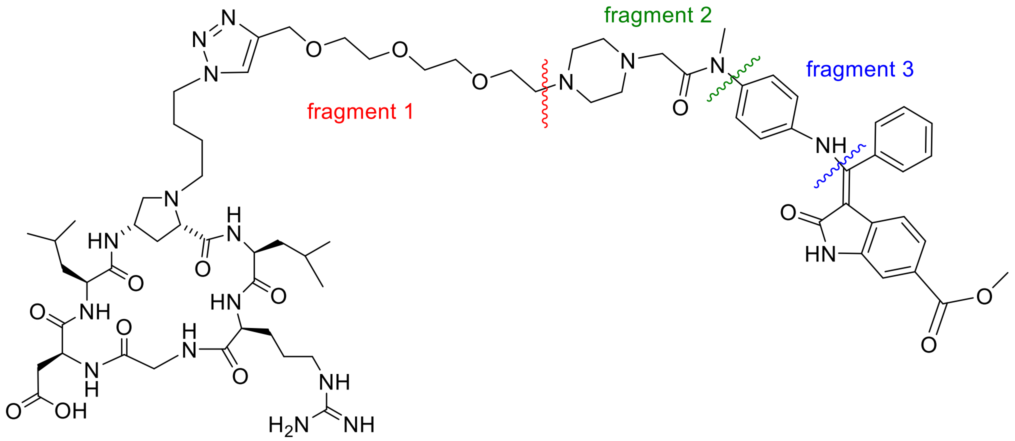

Figure S5. Indication of fragments 1-3 within the structure of conjugate PepNIN **1** used for docking calculations.

6. Statistical analysis

***In vitro* studies on human IPF Fibroblasts.** Expression of cell surface markers of mesenchymal or epithelial differentiation as well as αvβ6 integrin receptor was assessed in IPF-derived cell populations isolated from at least three different patients and one representative analysis is presented. Fluorescence intensity analysis in the internalization studies was conducted in IPF-derived cell populations isolated from at least three different patients, and one representative result is shown. Western blotting analysis of TGF-β1 production and ERK1/2 phosphorylation were assessed in three different patients. One representative result is shown. Densitometric data were analyzed using GraphPad Prism version 4 (GraphPad Software Inc., San Diego, CA). To evaluate statistically significant differences between group means across the different treatment conditions, a two-way ANOVA (Analysis of Variance) was conducted. A Tukey's Honestly Significant Difference (HSD) post-hoc test was then applied.

***In vivo* and *ex vivo* studies.** Statistical analyses were conducted using Prism 10 software (GraphPad Software Inc., San Diego, CA). Data are presented as mean±SEM. A t-test was used to compare fluorescence signals and gene expression results obtained from Saline and BLM groups. For the other parameters, One- or Two-way analysis of variance (ANOVA) was performed, followed by Dunnett's multiple comparisons post hoc tests. Normality of data was assessed using the Shapiro–Wilk test, supplemented by visual inspection of QQ-plots. The Pearson correlation coefficient (r^2) was calculated when correlating fluorescence with μCT parameters (e.g., %Poorly-aerated tissue). A p-value of < 0.05 (*) was considered statistically significant for all tests.

7. References

(1) Bugatti, K.; Andreucci, E.; Monaco, N.; Battistini, L.; Peppicelli, S.; Ruzzolini, J.; Curti, C.; Zanardi, F.; Bianchini, F.; Sartori, A. Nintedanib-Containing Dual Conjugates Targeting αVβ6 Integrin and Tyrosine Kinase Receptors as Potential Antifibrotic Agents. *ACS Omega* **2022**, *7*, 17658-17669.

(2) Choi, H.S.; Nasr, K.; Alyabyev, S.; Feith, D.; Lee, J.H.; Kim, S.H.; Ashitate, Y.; Hyun, H.; Patonay, G.; Strekowski, L.; Henary, M.; Frangioni, J.V. Synthesis and in vivo fate of zwitterionic near-infrared fluorophores. *Angew. Chem. Int. Ed.* **2011**, *50*, 6258–6263.

(3) Bugatti, K.; Bruno, A.; Arosio, D.; Sartori, A.; Curti, C.; Augustijn, L.; Zanardi, F.; Battistini, L. Shifting Towards αVβ6 Integrin Ligands Using Novel Aminoproline-Based Cyclic Peptidomimetics. *Chem. Eur. J.* **2020**, *26*, 13468-13475.
